# Fundamentals and Applications of Focused Ultrasound-Assisted Cancer Immune Checkpoint Inhibition for Solid Tumors

**DOI:** 10.3390/pharmaceutics16030411

**Published:** 2024-03-16

**Authors:** Sepideh Jahangiri, François Yu

**Affiliations:** 1 Microbubble Theranostic Laboratory, Centre de Recherche du CHUM, Montreal, QC H2X 0A9, Canada; 2 Faculty of Medicine, University of Montreal, Montreal, QC H3T 1J4, Canada; 3 Department of Radiology, Radiation Oncology and Nuclear Medicine, University of Montreal, Montreal, QC H3T 1J4, Canada

**Keywords:** microbubble cavitation, tumor microenvironment immune phenotype, immunotherapy

## Abstract

Despite spectacular clinical successes across several cancer types, immune checkpoint inhibition is effective only in subgroups of patients and suffers from significant systemic toxicities, highlighting the need to understand and locally overcome the mechanisms of therapeutic resistance. Similarly to other therapeutics, immunotherapies face delivery challenges (for example, antibodies need to reach their targets) and immunological barriers that are unique to solid tumors and their microenvironment. Interestingly, focused ultrasound (FUS), with or without microbubbles, which has been shown to enhance gene and drug delivery, notably in oncology, has been recently found to trigger immunological responses. In recent years, there has been a strong emphasis on understanding the biological and immunological effects of FUS for cancer therapy, and FUS is now emerging as an approach that can improve cancer immunotherapy. We herein review: (1) the immunological barriers implicated in ICI resistance; (2) the fundamentals of FUS +/− MB and the current knowledge on leveraging FUS +/− MB bioeffects for improving ICI therapy efficacy; (3) the immune profile of tumor models that have been successfully treated with FUS and ICI; and finally, (4) we discuss the challenges ahead for translating FUS and MB treatments to the clinic, highlighting the exciting perspectives for this new research area.

## 1. Introduction

Onco-immunotherapies, and specifically immune checkpoint inhibition (ICI), can restore the ability of the host’s immune system to fight cancer. ICI first emerged in the clinic for patients with melanoma, with a 26% overall response rate [1,2]. Monotherapy or combination therapy is now used in first-line therapy for advanced melanoma, non-small cell lung cancer [3], head and neck squamous cell carcinoma [4], and renal cell carcinoma [5]. However, ICI is not successful in a significant proportion of patients, nor in refractory cancer types. To improve immunotherapy efficacy and reduce systemic toxicities, there is a need to understand and find ways to overcome the mechanisms of therapeutic resistance.

Focused ultrasound (FUS) is an image-guided, non-invasive therapeutic modality that uses ultrasound waves to target tissues. Recently, FUS has been shown to elicit immunomodulation properties that could enhance immunotherapy [6]. Depending on the FUS parameters, adjuvant FUS can damage a targeted tumor (cytotoxic effects) or modulate the immune responses in a tumor, potentially overcoming some immune barriers to cancer therapy. The rationale builds on known bioeffects from blood–brain barrier (BBB) opening [7], ablative high-intensity focused ultrasound (HIFU) [8], and drug delivery [9] literature. For example, FUS can temporarily disrupt the BBB, delivering therapeutic agents, such as ICIs, directly to the tumor site [10]. Other studies have shown that FUS can increase immunogenic cell death and antigen presentation to turn an immunosuppressive (cold) tumor microenvironment (TME) into an inflamed (hot) TME [11].

In this review, our objective was to survey and categorize anticancer FUS studies, emphasizing the immunological barriers of tumors, the biological and immunological effects of FUS, and the heterogeneity of tumoral immune profiles. We thus begin this review with a short background on cancer development from an immune perspective (Section 2). Immune barriers within the TME that foster tumor growth and prevent an effective immune response are briefly discussed in Section 3. The bioeffects and immune effects of the major FUS regimes are reviewed in Section 4. In Section 5, we review the immune profiles of tumor models that have been treated with FUS to portray the state of the art. Finally, the current and potential clinical challenges and limitations of FUS + ICI are discussed in Section 6.

## 2. Cancer from an Immune Point of View and Immune Check Point Inhibition

From an immune perspective, cancer results from a failure of the immune system to recognize and eliminate abnormal/mutated cells. The immunoediting theory describes cancer as a dynamic process in which the immune system continuously interacts with the mutating cells and influences their fate [12,13]. Initially, in the elimination phase, T cells and natural killer (NK) cells can recognize and eliminate abnormal cells. However, over time, the immune positive selection pressure eventually drives the escape of a subgroup of mutant cells from immune surveillance, allowing their proliferation. Cancer cells typically achieve immune escape by altering their immunogenicity (mostly by downregulating MHC-I receptors resulting in antigen masking) and establishing an immunosuppressive TME. Thus, cancer immunotherapy consists of (re-)activating the immune system for identifying and eradicating tumor cells.

The discovery of programmed cell death-1 (PD-1 and its ligand, PD-L1) and cytotoxic T lymphocyte-associated protein 4 (CTLA-4) revolutionized cancer immunotherapy [14]. PD-1 and CTLA-4 are co-inhibitory receptors that normally function as breaks for the adaptive immune response, preventing damage to normal healthy tissues (autoimmune diseases) and leading to immune tolerance [15]. Signaling through these pathways contributes to the regulation of initial T cell activation, fine-tuning of T cell fate and functions, T cell tolerance, and return to immune homeostasis [16]. Hence, for an efficacious anticancer immune response, effector T cells must overcome immune checkpoint inhibitory signaling to exert their full functions. Currently, mono/combination therapy with pembrolizumab (commercial aPD1), is the first-line treatment for melanomas [17], metastatic triple-negative breast cancer [18], non-small cell lung cancer [19,20], advanced urothelial carcinomas [21], and cervical cancer [22]. The FDA approved ipilimumab (aCTLA-4) in 2011 for advanced melanoma [23]. New checkpoint pathways are being discovered (CD39, CD73, LAG3, TIM3, NKG2, etc.) [24], promising to increase response rates by combining non-redundant pathways. Increasing the proportion of responders to ICI is an active area of research because a complete understanding of the mechanisms of resistance to ICI remains elusive.

## 3. Immunological Barriers and TME Immune Profile

Immunological barriers in tumors refer to the various obstacles or mechanisms cancer cells use to evade the immune system. Tumors compromise immune responses by preventing T-cell activation, function, and survival. Firstly, cancer cells undergo metabolic reprogramming, including TME acidosis, heightened activity of indoleamine 2,3-dioxygenase (IDO), and an augmented adenosinergic pathway (Section 3.1). Increased IDO activity and adenosine (Ado) are important modulators of immune suppression. Secondly, immune suppressive chemokines, like transforming growth factor beta (TGF-β) and interleukin 10 (IL-10), exert their inhibitory effects on immune cells, fostering an immunosuppressive TME (Section 3.2). Another key immunological barrier is an impaired tumor mutational burden (TMB) and tumor-associated antigens (TAAs), which dramatically shape the TME immune profile. These tumors with low TAA and TMB levels typically do not respond well to immunotherapy, and they are reviewed in Section 3.3. Ultimately, the combination of all these factors leads to distinct patterns known as inflamed, excluded, and desert tumor immune phenotypes (Section 3.4). Understanding the dynamic interplay between immune barriers and TME immune profiles is crucial for developing innovative therapeutic approaches to overcome these challenges and to optimize the efficacy of ICI therapy in different tumor contexts.

### 3.1. Metabolic Reprogramming: Adenosinergic Signaling

Metabolic reprogramming is intricately linked to cancer cell growth. Orchestrated by various factors such as oncogenes, tumor suppressor genes, growth factors, and alterations in the TME, this reprogramming leads to resistance to traditional therapies. In our exploration, we narrow our focus to adenosine triphosphate (ATP) due to the reported impacts of FUS on this molecule. ATP and Ado are critical metabolic and immune regulators that modulate TME immunosuppression. Extracellular ATP (eATP) levels are higher in the TME (hundreds of micromolar) compared to physiological concentrations in the nanomolar range in healthy tissue. eATP concentration is regulated by cell surface ecto-nucleotidases. CD39 hydrolyzes ATP to ADP and AMP, whereas CD73 degrades AMP to Ado [25]. CD39 and CD73 are overexpressed in the hypoxic TME, which suggests their implication in tumor progression and immune suppression [26]. Ado is an immune suppressor binding to its receptors in T and B cells, DCs, and NK cells. The activation of Ado receptors inhibits proximal TCR signaling, as well as CD28 co-stimulation and IL-2R signaling, which are critical for T-cell activation, survival, and cytokine production [27,28,29]. This results in impaired T-cell function and reduces immune responses. Additionally, Ado binding increases the expression of immunosuppressive cytokines like IL-10, Foxp3, TNF-α, and IL-6, as well as co-inhibitory receptors such as PD-1, CTLA-4, LAG3, and TIM3. The upregulation of these co-inhibitory receptors is associated with the differentiation of regulatory T cells (Tregs) [30] and the exhaustion of effector T cells, a state of functional impairment that occurs during chronic infections or cancer [31,32,33].

### 3.2. Immunosuppressive Cytokines: TGF-β and IL-10

Immunosuppressive cytokines released from tumor cells or suppressive immune cells (such as Tregs, MDSCs, and TAMs) are key mediators of immune escape. TGF-β and IL-10 are overexpressed in cancers and impose immunosuppression by reducing the expression of effector cytokines associated with Th1, Th2, and Th17 subsets. This means IL-10 and TGF-β can suppress CD4^+^ T cell production of pro-inflammatory cytokines such as IFN-γ, IL-2, and IL-17 [34].

TGF-β suppresses cytotoxic T cells (CTLs) and NK cells and abrogates DC antigen presentation [35]. TGF-β in a positive feedback loop drives Treg differentiation; however, the presence of IL-6 or IL-21 can inhibit Treg differentiation and instead promote the differentiation of Th17 cells. This phenomenon is known as cytokine-dependent Treg/Th17 plasticity and is thought to be important in maintaining a balance between Treg and Th17 cell populations in the TME [36]. Cancer stem cells, Tregs, regulatory B cells, TAMs, MDSCs, and cancer-associated fibroblasts mediate the overexpression of TGF-β in the TME. Moreover, hypoxia stimulates the overexpression of TGF-β within the TME. Breathing supplementary oxygen can potentially convert the immunosuppressive TGF-β-enriched TME to a more normal stroma [37].

IL-10 is known to suppress the production and activity of pro-inflammatory cytokines such as IL-17, IL-6, and IL-12/23, promote the differentiation and function of anti-inflammatory Treg cells, and inhibit the activity of macrophages and Th17 cell responses [38]. IL-10 induces immune tolerance in T cells by selectively inhibiting TCR activation (CD28 co-stimulatory signaling dependency). However, T cells with strong TCR activation (independent of CD28) are not affected by IL-10 [39]. Additionally, IL-10 downregulates antigen-presenting cell (APC) function and upregulates the expression of CTLA-4.

Both TGF-β and IL-10 have bi-directional functions, meaning that in the pre-neoplastic state or the early stage of carcinogenesis, they act as tumor suppressor genes that inhibit cell proliferation and induce apoptosis [38,40]. Several studies have provided evidence that deficient IL-10 signaling promotes tumor development. In humans, IL-10 deficiency is associated with an increased risk of developing lymphoma [38,40]. Persistent IL-10 and TGF-β signaling, on the other hand, introduce selective pressure to overcome the tumor-suppressive effects of TGF-β or IL-10 [41]. This is known as the paradox of TGF-β and IL-10 and is associated with genetic mutations in the proteins involved in these pathways or changes in the TME [42]. At the same time, TGF-β signaling in the stromal cells within the TME can promote cancer progression and immune escape.

### 3.3. Genomic Instability, Tumor Mutational Burden, and Tumor-Associated Antigens

Genomic instability is defined as the tendency of cancer cells for increased genetic alterations (DNA mutation and chromosomal rearrangements) during cell division. Genomic instability results from defects in DNA repair systems, resulting in the accumulation of mutations in cancer cells. Tumor-associated antigens (TAAs), tumor mutational burden (TMB), and microsatellite instability (MSI) are measures of genomic instability that have been associated with increased immunogenicity and better responses to immunotherapy [43]. TMB and TAAs are both related to genomic alterations but represent different aspects of tumor biology. TAAs refer to antigens that are overexpressed or aberrantly expressed by cancer cells. TAAs are recognized by the immune system and can elicit an immune response, although they may not be as immunogenic as neo-antigens generated by a high TMB [43].

TMB refers to the total number of mutations present in the DNA of a tumor cell. High TMB can lead to the production of neo-antigens [43,44]. Microsatellites are DNA elements consisting of repeated sequences of 1–6 nucleotides. Microsatellite instability (MSI) is defined as the generation of alternate-sized repetitive microsatellites [45]. A small subset of CRC patients (15%) exhibit high MSI and have an inflamed TME, whereas the majority of CRC patients (85%) are microsatellite stable (CRC-MSS) [46]. Tumors with high TMB and an inflamed TME, including melanoma and locally advanced urothelial cancers, are good responders to ICI therapies [43,47,48,49,50,51,52]. Tumors with high TMB and high MSI, including metastatic bladder cancer, NSCLC, and CRC-MSI, are usually good responders to ICI therapy. In contrast, tumors with high TMB but without inflammation (small cell lung cancer (SCLC) and bladder cancer) and tumors with inflammation but without TMB (renal cell carcinoma, hepatocellular carcinoma (HCC), gastric cancer, head and neck cancers, and triple-negative breast cancer (TNBC)) are low responders to immunotherapy [43]. Recent research has suggested that combining TMB and PD-L1 expression may provide a more accurate prediction of response to immunotherapy than either biomarker alone [43].

The successful anti-tumor immune response process starts with TAA recognition by APCs. Subsequently, APCs activate effector immune cells, such as CTLs, initiating anti-tumor responses. However, the journey of effector cells to the tumor site and their infiltration into the tumor tissue can be challenging in the hostile TME. If the effector cells reach the tumor site, they must recognize and bind to the TAAs on the tumor cell surface. This recognition triggers the effector cells to attack and kill the tumor cells. As tumor cells are destroyed, they release more TAAs that APCs can process. This, in turn, activates more effector cells, enhancing subsequent anti-tumor activity.

### 3.4. TME Immune Profiles and Resistance to ICI Therapy

**Figure 1:**
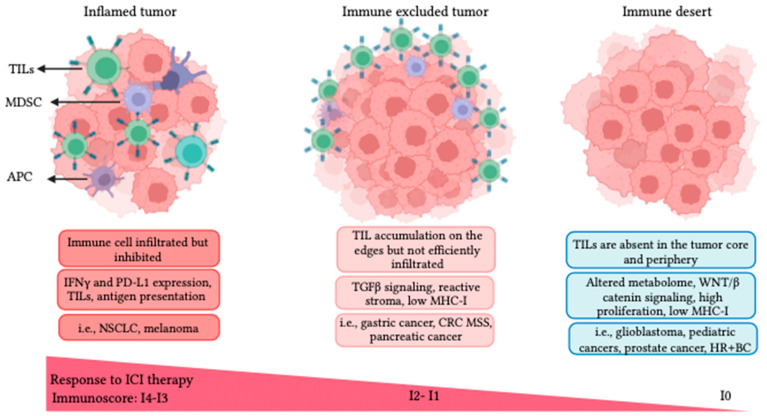
Tumor immune phenotypes defined by the lymphocyte presence, activation, and distribution in the TME. Inflamed tumors are infiltrated by TILs, present a high expression of IFN-γ and PD-L1, and typically respond to ICI. Immune-excluded tumors restrict TILs to the tumor periphery, with a prevalence of myeloid and suppressor cells presenting strong TGF-β signaling with reduced antigen presentation. Immune desert tumors exhibit a complete loss of immune activity (no TILs), and present an altered metabolome, resulting in quick cell proliferation [57]. Some tumor examples for each immunophenotype are shown in the figure. ***Acronyms***: NSCLC: non-small cell lung cancer; CRC: colorectal cancer; HR^+^ BC: hormone receptor positive breast cancer.

For some cancers, ICI alone can restore anticancer immune responses. However, this is not the case across cancer types or even across patients within a cancer type. The TME immune profile has been proposed to stratify candidate patients for immunotherapy. The immunoscore describes the level of two infiltrated lymphocyte populations: (1) all tumor-infiltrating lymphocytes (TILs = CD3^+^) and (2) CTLs (CD8^+^) and their spatial distribution in the tumor [53]. The immunoscore ranges from I4 (highly lymphocyte infiltrated) to I0 (absence of lymphocytes) and classifies cancers into three subcategories, namely ***inflamed*** with I4-I3 (hot), ***immune-excluded*** with I1-I2 (low and moderate immune cell infiltration), and ***immune desert*** with I0 (cold), as depicted in Figure 1 [43,54,55].

***Inflamed tumors*** are typically linked to favorable responses to ICI therapy. Histopathological examination of inflamed tumors reveals a distribution of TILs in the tumor parenchyma in close contact with cancer cells. However, the expression of immune cell exhaustion markers, like PD1/PDL1, can inhibit the activation of infiltrated effector immune cells [43,54]. These tumors also generally present a high genomic instability, leading to a high TMB and elevated TAA levels. It has been reported that the fraction of patients who have a higher TMB and pre-existing anti-tumor immune responses have a greater survival benefit from ICI therapy [43,56]. Therefore, high TMB and TAA levels, genomic instability, an elevated presence of B cells, increased expression of IFN-γ, and low expression of TGF-β are biomarkers of inflamed tumors associated with better prognosis following ICI therapy [43,54]. NSCLC and melanoma are good examples of “hot” or inflamed phenotypes [43,50,51,52].

***Immune-excluded*** tumors are poor responders to ICI therapy. In histopathological examination, lymphocytes are typically found in the tumor margins with high TGF-β expression, enhanced myeloid presence, and angiogenesis [43]. Mariathasan et al. (2018) reported a key role for TGF-β in promoting the excluded TME phenotype. They have also shown that TGF-β suppression can sensitize immune-excluded tumors to ICI therapy, turn immune-excluded tumors to the inflamed type, and augment inflammation in the TME in EMT6 breast cancer animal models [58]. An increased myeloid population within the TME, low TMB, and low MHC-I are associated with the immune-excluded phenotype. MC38 and EMT-6 tumor models are examples of immune-excluded tumors in which MDSCs are prevalent and T cells are excluded from the tumor margins. Combination therapy of aTGF-β and aPDL1 in both MC38 and EMT-6 mouse models increased T-cell infiltration and distribution, provoked anti-tumor immunity, and inhibited tumor growth [58]. Pancreatic ductal adenocarcinoma, TNBC, and hepatocellular carcinoma are examples of immune-excluded cancers.

***Immune desert*** or “cold” tumors are usually resistant to ICI therapy. Immune desert tumors lack immune infiltration and antigen presentation and exhibit high proliferation rates. This phenotype is characterized by the absence of T cells, low TMB, and inadequate antigen presentation. The low expression of MHC-I leads to poor T-cell priming, further contributing to the limited immune response in these tumors. Chemokine secretion of immune attractants like CXCL1, CXCL2, and CCL4 that recruit DCs are markedly downregulated, which further prohibits T-cell priming and infiltration in immune desert tumors [43]. Accumulating evidence demonstrates aberrant WNT/β-catenin signaling favoring malignant transformation and promoting immune escape and ICI resistance [59]. Additionally, unrestrained cancer cell proliferation results in increased hypoxia and acidosis, which alters T-cell metabolism, TCR engagement, function, and proliferation [57]. Early-onset cancers, like pediatric and juvenile malignancies, and aggressive adulthood cancers like glioblastoma, small cell lung cancer (SCLC), prostate, and HR^+^ breast cancers are recognized as cold tumors [43,54]. The potential relation of these immune profiles with FUS therapy will be discussed later in this paper.

## 4. Focused Ultrasound (FUS) Modalities for Cancer Therapy

**Figure 2:**
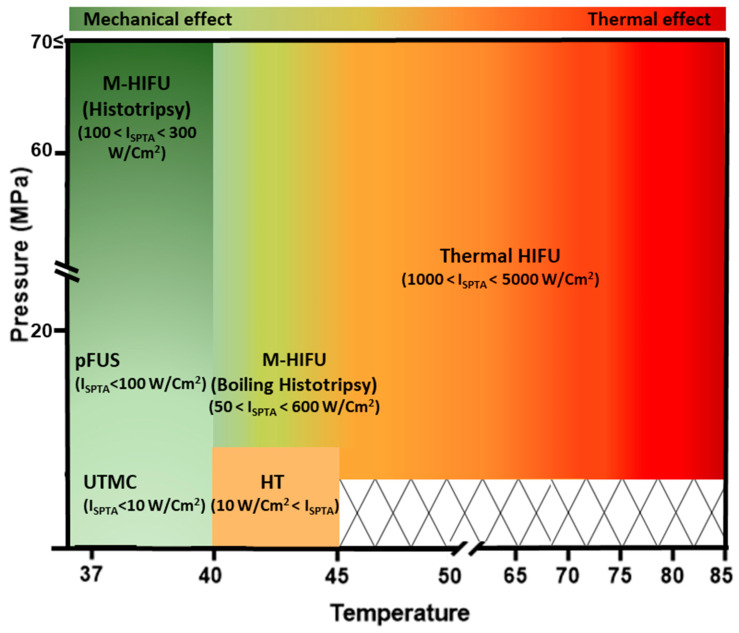
An overview of the relationship between I_SPTA_, temperature, pressure, and thermal damage for different FUS modalities. There is minimal thermal damage below the thermal threshold. Thermal damage is strongest for T-HIFU, which has the greatest I_SPTA_. ***Acronyms***: I_SPTA_: spatial peak temporal average intensity; T-HIFU: thermal HIFU; M-HIFU: mechanical HIFU; pFUS: pulsed focused ultrasound; UTMC: ultrasound-targeted microbubble cavitation.

Diagnostic ultrasound imaging is an established imaging modality for disease diagnosis and management. Ultrasound can also be used therapeutically, as supported by several clinical trials evaluating the efficacy of ultrasound, with or without microbubbles (MBs), to open the blood–brain barrier (NCT03712293, NCT02343991, NCT05733312, NCT03714243, and NCT05317858) for neurodegenerative disease therapy or in combination with chemotherapy (NCT03477019) or immunotherapy (NCT03237572, NCT04116320, and NCT04021420) for cancer treatment and management. FUS treatment offers a novel targeted, non-invasive, non-ionizing treatment that can replace or complement traditional therapies (surgery, chemotherapy, radiotherapy) to improve patient quality of life and survival [60]. FUS takes advantage of mechanical sound waves, typically >20 kHz that can be focused deep (~10 cm) into the body. FUS modalities used in cancer therapy differ by their sonication parameters and the type of stress induced. We grouped them into five categories, namely ***thermal ablation high-intensity focused ultrasound (T-HIFU)***, ***mechanical ablation HIFU (M-HIFU)***, ***hyperthermia (HT)***, ***pulsed focused ultrasound (pFUS)***, and ***ultrasound-targeted microbubble cavitation (UTMC)***. T-HIFU and M-HIFU are ablative, whereas HT, pFUS, and UTMC are typically non-ablative. HIFU treatment usually leads to more necrosis than apoptosis, while UTMC causes more apoptosis than necrosis [6,61]. Beam characteristics and mechanisms of action of each FUS modality are elaborated in Section 4.1, Section 4.2, Section 4.3, Section 4.4 and are summarized in Figure 2 and Table 1.

### 4.1. Thermal Ablation HIFU (T-HIFU)

T-HIFU is FDA-approved for treating several solid malignancies (e.g., pancreas, bone, liver, prostate, breast, and kidney) [80]. For T-HIFU, near continuous (DC% close to 100%) high-intensity US waves are used at a frequency of ~1–8 MHz, a spatial peak temporal average intensity (I_SPTA_) > 1000 W/cm^2^, and a high pressure of 3–70 MPa to heat the tumor. The energy of the US beam is absorbed by the targeted tissue, heating it to 60–85 °C. T-HIFU leads to coagulative thermal necrosis. Surrounding tissues around the focal spot, which receive lower temperatures, typically become apoptotic [81].

T-HIFU achieves primary tumor control but is not always effective at generating abscopal effects, suggesting that HIFU may fail to prime an adaptive anti-tumor immune response. The high thermal stress induced by HIFU destroys the vasculature and tissue structure, which may limit the ability of immune cells to reach the tumor site and support antigen presentation [6]. T-HIFU releases many immune-activating molecules such as TAAs, damage-associated molecular patterns (DAMPs), and heat-shock proteins (like HSP73, HSP72, HSP70, HSP60 and HSP27). These immunostimulatory molecules are recognized by APCs (i.e., DCs) and initiate leukocyte recruitment and infiltration to the TME [82]. Activated DCs in the HIFU-ablated region secrete IL-12 and IFN-γ to recruit and activate CTLs. CTLs release TNF-α and IFN-γ, further stimulating the anti-tumor immune responses [83]. T-HIFU, despite quickly destroying tumor integrity and shrinking the tumor mass, is yet unable to markedly provoke immune stimulation within the TME due to the massive coagulative necrotic region. T-HIFU monotherapy is insufficient for stimulating adaptive immune responses [6,84]. Another limitation of this technology could be that elevated temperatures may denature released TAAs and thus dampen the anti-tumor immune response. Interestingly, combining ICI therapy with T-HIFU ablation could foster systemic and long-term anti-tumor immunity in tumor models prone to distal metastasis or recurrence [6,84,85]. The tissue intrinsic characteristics, like the level of stromal condensation or the ratio of residual mesenchymal cells to tumoral cells, determine the success of HIFU-mediated anti-tumor immune responses, which can determine the extent of immune priming or tolerance. Therefore, HIFU parameters needed to be optimized for every tissue [6,76].

### 4.2. Mechanical Ablation HIFU (M-HIFU)

M-HIFU refers to ablative boiling histotripsy and non-thermal histotripsy modalities of FUS. Histotripsy is derived from the Greek “histo”, meaning tissue, and “tripsy”, meaning to crush or grind. During a histotripsy procedure, a high-pressure US beam is focused on a small area of tissue, creating bubbles of gas. These bubbles expand and collapse rapidly, leading to micro-jetting, streaming, and shear stress that mechanically pulverize the tissue [6,86,87]. It typically uses short pulses of US waves (micro- to millisecond pulses) with a low duty cycle (0.1–4%), frequencies below 3 MHz, and I_SPTA_ > 50 W/cm^2^ at very high pressures (10–80 MPa).

M-HIFU can fractionate tissue into sub-cellular fragments with or without thermal damage. Boiling histotripsy refers using longer (milliseconds) pulses, which induce both mechanical and thermal ablation in the targeted tissue [88]. Non-thermal histotripsy uses very short pulses of HIFU that create rapid expansion and collapse of gas bubbles without causing thermal effects [70]. Boiling histotripsy can destroy cells and tissues at a faster rate than histotripsy; however, it also increases the risk of thermal damage to surrounding healthy tissue. It is important to note that the choice of pulse duration depends on the type of tissue and the desired outcome, and it represents a delicate balancing act between effective fragmentation and minimal thermal damage [89]. In contrast to T-HIFU, histotripsy produces intact antigens (non-thermally damaged) in a mechanically-dominated mechanism, which is believed to improve antigen presentation and anticancer immunity. It is also more precise than T-HIFU and leaves the surrounding normal tissue undamaged since there is no thermal diffusion [6].

Lesions produced by boiling histotripsy exhibit microhemorrhage, immune cell infiltration, and a homogenate of cellular debris [81,90]. Released TAAs are captured by APCs and migrate to the TDLNs and the spleen, where APCs are in close contact with T cells to activate them. Released DAMPs include calreticulin [91,92,93], HSP70 [93,94], and the local and systemic release of HMGB1 [92,93], inducing cancer cell death [95]. Calreticulin is an endoplasmic reticulum-associated protein that is exposed on the surface of dying tumor cells after non-thermal histotripsy treatment [92]. This can act as an “eat-me” signal, promoting the clearance of tumor cells by immune cells. Following histotripsy, the elevation of IFN-γ is the most consistently reported cytokine by several studies [92,93,96,97]. The upregulation of IL-1, IL-2, IL-6, IL-10, IL-13, IL-18, and TNF-α is reported to indicate an immune response in the TME [92,93,96].

Following non-thermal histotripsy, innate immunity (NK cells, DCs, and macrophages) is recruited to the tumor [92,98]. In a melanoma model, an intratumoral increase in NK cells, DCs, macrophages, and neutrophils was found 10 days post-treatment. Interestingly, infiltrative CTLs were also increased at both primary tumor and metastatic lesions, indicative of an adaptive immune anti-tumor response [92]. Histotripsy-mediated CTL infiltration induces immunogenic cell death through ferroptotic cancer cell death in melanoma and hepatocellular murine models [98]. In a thymoma mouse model (EL4), boiling histotripsy was compared with T-HIFU [99]. In boiling histotripsy, microhemorrhages were found in a narrow transition zone between the disintegrated sonicated region and the viable non-focal tumor tissue. The infiltration of granulocytes and macrophages was markedly increased 4 days after treatment. However, in the T-HIFU group, no immune cell infiltration was identified [99].

### 4.3. HIFU-Induced Hyperthermia (HT)

In HT, the US duty cycle can be adjusted (0.5–100% range) at frequencies of 0.2–3 MHz, I_SPTA_ > 10 W/cm^2^, and intermediate pressure levels of 1–5 MPa. In HT, tissue temperature is increased mildly to 40–45 °C but is maintained for a longer duration (30 to 90 min). HT is most effective in low-perfused tumor tissue that cannot dissipate heat [100]. Typically, HT is associated with drug delivery using thermosensitive drug carriers, e.g., thermosensitive liposomal doxorubicin in liver cancer patients [81,90], murine breast cancer models [75], or murine ovarian carcinoma [101]. Other pleiotropic effects of HT include cell cytoskeleton distortions [102], protein and DNA damage leading to cell cycle arrest, DNA repair abrogation, and ultimately apoptosis [103]. The putative biological mechanisms are mediated by HSP upregulation, p53 activation, mitochondrial injury, and caspase 2 activation. Also, HT induces vasodilation that increases blood perfusion and oxygenation to the TME, reducing hypoxia, acidosis, interstitial tumor pressure. HT markedly inhibits the DNA repair system and thus can sensitize cancer cells to chemotherapy [103]. The effects of HT are strongly dependent on the tumor type, temperature elevation, and exposure time [104]. Gouarderes et al. (2020) indicated that HT could loosen the tight structure of the extracellular matrix (ECM) and disrupt the connective tissue, enabling drug penetration 2.5 times greater than that in untreated tumors [105], reducing the interstitial fluid pressure, and increasing the number of infiltrated CAR-T cells [106].

From an immune perspective, HT stimulates multiple pathways of innate and adaptive anti-tumor immune responses. HT is associated with increased DAMPs in the TME, including, eATP release, surface-expressed calreticulin, HMGB1, HSP90, and HSP70 [107]. The release of DAMPs within the TME activates quiescent DCs to recruit inflammatory immune cells to the site, resulting in CTL activation by inducing granzyme B expression and increasing IFN-γ, IL-10, and IL-6 secretion [6]. Thermal stress has been shown to differentiate mature DCs through increased levels of HSP90. Tumor-specific CTLs and NK cell-mediated anti-tumor immunity are stimulated by HT [108,109]. HT acts directly on both lymphocytes and the vascular endothelium to increase lymphocyte diapedesis and migration by upregulating LFA-1 and ICAM-1, respectively [110,111].

Recent studies proposed that both HIFU thermal modalities—T-HIFU and HT—can enhance TAA and CTL infiltration but are not sufficient to increase ICI efficacy in animal models [75,85,112,113]. Therefore, additional stimuli such as immunoadjuvants or chemotherapies may be necessary to augment cross-presentation and cross-priming mediated by APCs.

### 4.4. Pulsed FUS (pFUS)

pFUS employs non-ablative short pulses to induce acoustic cavitation and acoustic radiation forces. pFUS typically uses DC% = 0.5–20% at low frequencies (<3 MHz) with relatively high I_SPTA_ (<100 W/cm^2^) and a peak negative pressure of 0.1–5 MPa [76,77,78]. Pulse intervals minimize the effects of temperature elevation (<5 °C), lower energy deposition, and allow tissue cooling [114]. pFUS can be used for enhanced drug delivery (doxorubicin) to KPC-bearing mouse models [115]. Another study used low-powered pFUS (5 to 20 W/cm^2^) to break down the integrity and stiffness of the ECM, allowing deep tissue transportation of nanoparticles in a tumor model of lung adenocarcinoma (A549 cell line). Histological staining confirmed pFUS-mediated ECM remodeling without vascular damage [116].

pFUS can induce apoptosis, reduce viability in cancer cells, and increase TIL density in tumor tissue [117]. Bandyopadhyay et al. (2016) reported that pFUS can increase antigen presentation, reverse the tumoral T cell tolerance by increasing cytokine secretion, reduce the anergy-related gene expression profile, and increase the percentage of activated/matured DCs. In this study, pFUS treatment followed by ablative M-HIFU induced a complete rejection in 80% of B16-bearing mice, markedly improved recurrence-free survival, and reduced local or distal metastasis, indicative of abscopal immune activity [118]. In contrast to HIFU, pFUS stimulates inflammatory responses with limited cellular damage [114]. pFUS induced a spike of several pro-inflammatory cytokines (TNFα, IL-1α, IL-1β, IFN-γ, GMCSF) on day 1 post-pFUS and returned to baseline on day 3. pFUS also induced the expression of ICAM-1 and VCAM-1 at day 0 and day 1 [114]. Aydin et al. (2019) also studied the proteomic profile in the TME following pFUS treatment. They reported DNA strand breaks at peak negative pressure (PNP) > 6 MPa and a downregulation of immunosuppressive response at PNP > 4 MPa in the B16 tumor model 24 h post-sonication [76]. Cohen et al. (2021) studied anti-tumor immune responses in B16 and 4T1 models. Following pFUS, there was a 75% concordance for anti-tumor cytokines and inflammatory markers (including TNFα, IL-1a, IL-1b, IL-17, IL-6, and VCAM-1), indicating a TME shift toward a hot TME. However, the anti-tumoral cellular immune response was only found in the 4T1 model. Therefore, the anti-tumor immune phenotype following pFUS is strongly dependent on the tumor type [77]. Moreover, their transcriptomic data revealed that over 12 h post-treatment, the KRAS and EMT signaling pathways were upregulated in B16 tumors, indicating proliferative and aggressive features, while these pathways were downregulated in 4T1 tumors consistently, with greater tumor growth inhibition in the 4T1 model [77]. This study highlighted that intrinsic features of the primary tumor, like desmoplastic and ECM characteristics, affect the strength of the generated response following FUS treatment and exemplify the necessity of fine-tuning FUS parameters for every tumor type.

### 4.5. Ultrasound-Targeted Microbubble Cavitation (UTMC)

**Figure 3:**
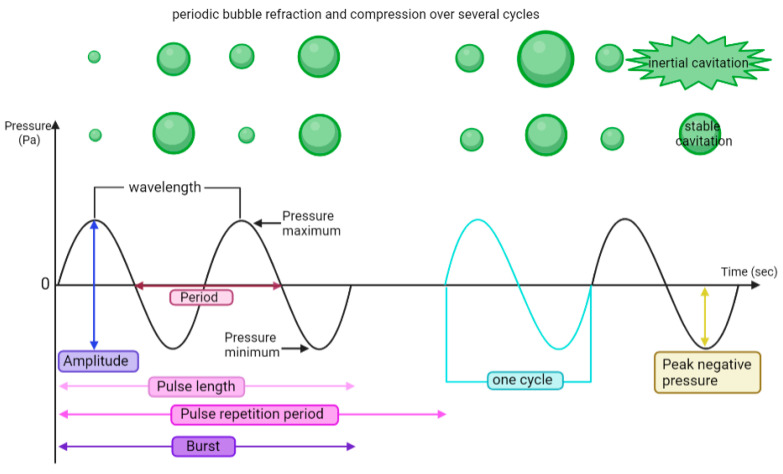
Ultrasound parameters and periodic bubble oscillations over several cycles. Basic US parameters that govern bubble behavior are described. Duty cycle is defined as the percentage of ON time divided by the total time (pulse length/pulse repetition period). Pulse repetition frequency (PRF) is the inverse of the pulse repetition period (PRF = 1/PRP). The mechanical index characterizes the extent of cavitation-induced mechanical bioeffects and is calculated as (MI = PNP(MPa)/√F(MHz)). I_SPTA_ (spatial peak temporal average intensity) = DC% × (P^2)/(2 × density × sound speed)) describes the time-averaged US power per unit area.

UTMC uses low-intensity pulsed ultrasound at a DC% of 0.5–50%, frequencies 0.2–3 MHz, I_SPTA_ < 10 W/cm^2^, and low to moderate pressure (0.1–4 MPa) combined with a systemic injection of MBs [6,90]. MBs have been used as US contrast agents for diagnostic imaging for several decades and are making their way into the therapeutic field [119]. Current commercial MB formulations comprise a gas core (typically perfluorocarbon or sulfur hexafluoride) stabilized by an amphiphilic membrane (lipid, polymer, or protein). MBs undergo volumetric oscillations (stable cavitation) and/or violent collapses (inertial cavitation) when subjected to US (Figure 3). MB stable and inertial cavitation produces shear stress on the nearby cell membranes and vessel boundaries, which can lead to mechanical damage (either reversible or irreversible). The infusion of MB typically reduces the pressure threshold, causing bioeffects compared to pFUS or M-HIFU. Transient membrane opening is termed sonoporation and can be exploited for drug or gene delivery [81,87,120]. Microstreaming helps to push the drug through the tumor stroma, thereby facilitating therapeutics delivery through the transvascular–interstitial–intracellular barriers [121].

UTMC treatment alters cellular metabolism, including modifications in cell membrane potential [122], calcium signaling [123,124], purinergic signaling [125,126], MAPK (mitogen-activated protein kinase) activation, and mTOR (mechanistic target of rapamycin) activation [127] while suppressing ERK 1/2 signaling [128]. UTMC induces endoplasmic reticulum (ER) stress that is characterized by the accumulation of misfolded proteins in the ER [129].

UTMC can augment immune cell infiltration to the site of interest [9,106,130,131,132]. This approach has been reported to enhance drug concentration and improve therapy efficacy by 20–80% [119,133]. MB oscillation can break down tight junctions between vascular endothelial cells and compromise vessel integrity [119,134]. Therefore, UTMC-induced vascular permeabilization enhances drug delivery [130,135] in the cornea [136], gastrointestinal tract [137], skin [138], and neural system [139,140,141]. Heath et al. (2012) investigated the benefit of combined UTMC with cetuximab (an antibody targeting EGFR) in a mouse model of head and neck squamous carcinoma; they showed a 30% increase in cetuximab delivery following UTMC [135]. In parallel, Amate et al. (2020) reported that UTMC and the subsequent sonoporation on the vasculature system promoted local antibody concentration and extravasation in solid tumors [130].

Several groups have investigated the potential of UTMC cavitation in BBB opening for enhancing drug delivery or the homing of immune cell adoptive transfer (CAR-T cells, NK cells, and vaccine DCs) in brain tumor mouse models [139,142,143,144,145]. Meng et al. (2021) recently demonstrated enhanced trastuzumab delivery in four patients with progressive intracranial HER2^+^ brain metastases in a single-armed open-labeled clinical study (NCT03714243) using MRI-guided UTMC. Trastuzumab + UTMC (six treatments for every single patient) was reported safe without hemorrhage or edema. Importantly, an increased standardized uptake value ratio of 101 ± 71% and a 19 ± 12% decrease in tumor size were found. Their results demonstrate the first evidence in humans of non-invasive, non-ionizing, spatially targeted, and safe mAb delivery through the BBB using MR-guided UTMC [146].

Recently, UTMC has been reported to decrease tumoral IFP [147,148] and therefore increase drug delivery to head and neck squamous cell carcinoma in mice and anaplastic squamous cell carcinoma in rabbits [78,148]. UTMC with oxygen MBs can overcome hypoxia, increase tumor oxygenation to 20 mmHg, and radiosensitize an in vivo breast tumor model [149]. Ho et al. (2019) demonstrated that UTMC, with oxygen MBs and doxorubicin, induces tumor oxygenation, inhibits the HIF-1α/VEGF pathway, and normalizes vessel structure by increased pericyte coverage while also increasing doxorubicin concentration in the TME [150]. Therefore, UTMC treatment can enhance tumor perfusion, relieve hypoxia, and offer a promising strategy for addressing dysfunctional and tortuous vasculature to improve anti-tumor therapies. Nevertheless, the impact of UTMC on immune cell distribution within a disrupted ECM network, as well as the undetermined bioeffects resulting from sonoporation and/or microstreaming, are areas that require further investigation.

Given that UTMC can be adjusted to either shut down [151,152,153,154,155] or preserve tumoral perfusion, it becomes essential to distinguish the effect of flow on the immune response. Ablative UTMC, which shuts down flow, has been found to increase the expression of HSPs and their translocation to the cell surface, thereby enhancing NK cell-mediated cytolysis in 4T1 and TPSA23 tumors [156]. Additionally, Hunt et al. (2015) discovered that ablative UTMC increased the presence of mature CD45^+^/CD3^+^ immune cells in the TME [157]. On the contrary, non-ablative UTMC, in addition to increasing local drug concentration, enhances anti-tumor responses by inducing vascular inflammation (ICAM-1, VCAM-1) [158], facilitating CTL infiltration [90], decreasing cancer cell proliferation [158], and promoting immunogenic cell death [6]. UTMC induces the polarization of tumor-associated macrophages from M2 to M1 [159]. In a murine pancreatic ductal adenocarcinoma model, a single UTMC treatment increased tumoral cytoplasmic HMGB1 and enhanced antigen presentation by macrophages, CTLs, and CD4^+^ cells only in TDLNs at 2 days after UTMC, which resulted in reduced tumor growth [160]. However, most of these changes had subsided after two weeks, demonstrating the need for additional treatments or combination with other therapeutics.

Mechanical perturbation by UTMC increases DAMPs (eATP, HMGB1, HSP60, HSP70, and calreticulin), favors DC maturation (MHC-II and B7 proteins), and stimulates adaptive immune responses [118,126,160,161]. Kovacs et al. (2017) found that sterile inflammation was induced in the parenchyma following UTMC treatment for BBB opening. DAMPs (HSP70, TNF-α, IL-18, and IL-1 but not ATP or HIF1α) increased post-sonication, lasting 24 h [142]. Moreover, increased levels of cytokines, chemokines, trophic factors (CCTFs), and vascular inflammation (ICAM-1) potentiated the pro-inflammatory immune responses following BBB disruption. The differential gene expression following BBB/BTB opening at 6 and 24 h post-UTMC revealed an increased gene expression of pro-inflammatory markers, including TNF-α, IL-6, CXCL family members, and ICAM-1, which potentiate immune cell trafficking and activation [162]. Moreover, increased DC infiltration into the meninges and intracranial tumor lesion indicated a better antigen presentation (correlated with increased expression of TAP1 and TAP2) to effector immune cells, while DC level was not changed in TDLNs. In this study, despite elevated cytokine expression, TILs did not increase at 2 days or 4 days post-treatment [162]. The latter could be improved by repeating UTMC treatment as well as choosing a later time point (7 days instead of 2–4 days).

## 5. Immune Profiles, FUS Bioeffects, and ICI

The three main objectives for successful ICI therapy for solid tumors are (1) tumor regression, (2) TAA release leading to T-cell activation, and (3) pro-inflammatory cytokine release to inhibit immunosuppressive cells (Tregs and MDSCs) and activate effector cells. To have a robust anti-tumor response, effector T cells need to be activated via MHC-I ligation of APCs like DC to CD8^+^ receptors on CTLs [163]. Mechanical or thermal FUS modalities have been shown to increase tumor antigen presentation to activate effector T cells and induce direct immunological effects [6,164,165]. The different FUS modalities and their mechanisms of action in inducing an immune response are reviewed in this section, with a particular emphasis on the tumoral immune phenotypes and TMB.

**Figure 4:**
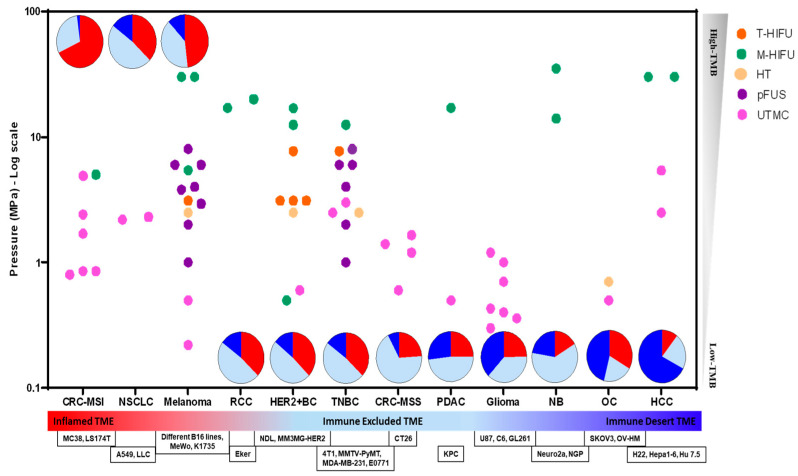
An overview of the relationship between immune phenotype (x-axis) and FUS modalities (color-coded and localized by pressure on the left y-axis) inducing anti-tumor immune responses in preclinical cancer models. Cancer types are also displayed in the form of pie charts representing their heterogenous clinical presentation as immune inflamed, immune-excluded, and immune desert fractions. Pie charts are localized vertically based on their TMB (right y-axis). **Methodology:** The immune phenotypes are adapted from [43] for melanoma, NSCLC, TNBC, RCC, PDAC, and CRC-MSS. HER2^+^BC is reported to have a similar phenotype to TNBC [167,168]. The immune phenotypes are determined through TIL infiltration proportions and immune transcriptome profiling in neuroblastoma [169], glioma [170], gene expression data from the Cancer Genome Atlas for OC [171], and TIL infiltration for CRC-MSI [46,172] and HCC [173]. **Acronyms:** CRC-MSI: colorectal cancer—microsatellite instable; NSCLC: non-small cell lung cancer; RCC: renal cell carcinoma; HER2^+^BC: HER2^+^ breast cancer; TNBC: triple-negative breast cancer; CRC-MSS: colorectal cancer—microsatellite stable; PDAC: pancreatic ductal adenocarcinoma; NB: neuroblastoma; OC: ovarian cancer; HCC: hepatocellular carcinoma; TMB: tumor mutational burden; T-HIFU: thermal HIFU; M-HIFU: mechanical HIFU; HT: hyperthermia; pFUS: pulsed FUS; UTMC: ultrasound-targeted microbubble cavitation.

A growing number of publications have reported encouraging results combining FUS and ICI therapy at the preclinical stage on cancer models with different immune profiles. These studies are summarized in Table S1 (Supplementary Data), and if they induced an anti-tumor immune response, they are graphically depicted in Figure 4. Based on Figure 4, melanoma (inflamed TME with high TMB) and TNBC (immune-excluded TME with low TMB) are the most extensively studied models. CRC-MSI (inflamed TME with high TMB), HER2^+^BC (immune-excluded TME with low TMB) and glioma (immune desert TME with low TMB) are the second-most studied. pFUS has been primarily studied in melanoma (inflamed TME and high TMB), and TNBC (immune-excluded TME and low TMB). UTMC has been studied in many models, but mostly in immune-excluded models (please note that MC38 is CRC-MSI but is immune excluded). T-HIFU is predominantly investigated in HER2^+^BC, while M-HIFU is frequently employed across different TME immune phenotypes.

The FUS + ICI combination can be split into approaches that: (1) shut down blood perfusion [85,166], either by T-HIFU or M-HIFU +/− MB treatment, or (2) improve blood perfusion [90,150], usually by HT, pFUS, and UTMC. Each modality exhibits different success rates in inhibiting tumor growth, overall survival rate, and response rate.

### 5.1. Ablative FUS + ICI

Silvestrini et al. (2017) demonstrated that priming with TLR agonist -CpG- and aPD-1 in a HER2^+^BC metastatic breast cancer model (NDL, immune-excluded TME, and low TMB) upregulates innate anti-tumor responses and alters infiltrated macrophage polarization after T-HIFU [85]. The follow-up study identified mechanistic potential differences between this combination and ICI monotherapy. First, there was a greater systemic antigen cross-presentation in the TDLNs in the combined treatment group vs. either monotherapy (16% vs. 10% or 0.1% of CD45^+^ cells, respectively). Second, the combined treatment polarized macrophages and DCs toward a CD169-expressing subset (phagocytosis) and enhanced tumoral release of IFN-I [174]. Sheybani et al. (2020) demonstrated that partial T-HIFU is not sufficient to elicit a robust T-cell response in a TNBC model (4T1, immune-excluded TME and low TMB) [112]. The combination of gemcitabine and the partial T-HIFU restricted tumor growth, but only minimal changes in intratumoral CTL were achieved. The addition of aPD-1 to T-HIFU + gemcitabine moderately improved growth inhibition when given before or after T-HIFU. Partial (2%) M-HIFU treatment on a large neuroblastoma model (Neuro2a, immune desert TME with low TMB) induced cellular and chemokine responses but was not sufficient to debulk the tumor and had no survival benefit [96]. The addition of aCTLA-4 and aPD-L1 to M-HIFU resulted in some complete tumor rejection and increased long-term survival from 0% (in either monotherapy) to 62.5% (in the combined treatment). M-HIFU increased NK cells in the spleen and TDLNs, systemic IL-2, IFN-γ, and DAMPs while decreasing CD4^+^ FOXP3^+^, IL-10, and VEGF-A at 24 h, 48 h, and 72 h post-treatment. Combined treatment with M-HIFU + aCTLA-4 + aPD-L1 significantly enhanced CTLs, DCs, Th cells, and Tregs in TDLNs and reduced systemic IL-10 [96]. M-HIFU and T-HIFU were compared in murine melanoma models (B16GP33, inflamed TME with high TMB) and hepatocellular carcinoma (Hepa 1–6, immune-excluded TME with high TMB). This study reported a stronger immune stimulation following M-HIFU treatment [92]. Additionally, M-HIFU was more effective in abscopal immune responses based on the number of pulmonary metastases. Moreover, immunogenic TAA was higher in M-HIFU and was correlated with calreticulin translocation and HMGB1 release. In this study, M-HIFU enhanced the efficacy of aCTLA-4 in both melanoma and hepatocellular carcinoma models [92]. Similarly, Abe et al. (2022) compared M-HIFU and T-HIFU in a breast cancer model, a TNBC model (E0771, immune-excluded TME with low TMB), and a HER2^+^BC model (MM3MG-HER2, immune-excluded TME with low TMB) [175]. They reported a more potent immune response and tumor growth inhibition in the M-HIFU-treated groups, with TAM polarization toward the M1 subtype in the M-HIFU group. This study reported that combining M-HIFU with aPD-L1 mediated superior immune responses, increased CTL and NK infiltration, and an abscopal effect. Fite et al. (2021) also compared M-HIFU and T-HIFU in combination with aPD-1 in a multi-focal breast cancer mouse model (NDL, immune-excluded TME with low TMB) [84]. M-HIFU and T-HIFU monotherapies provoked innate immune responses (increased NLRP3, Jun, MEFV, IL-6, and IL-1β) but no adaptive immune response. They found increased IL-6, IL-1β, MDSCs, and tumor regrowth. CTL infiltration into the TME was only found when T-HIFU was combined with aPD-1 [84].

Ablative UTMC treatment was combined with aPD-1 in a CRC-MSS model (CT26, inflamed TME with low TMB) [166]. Here, shutting down the blood perfusion induced more tumor necrosis and growth inhibition than when UTMC or aPD-1 was applied alone. Also, enhanced IFN-γ expression improved T cell activation. This group applied the same methodology of combined UTMC, aPD-L1, and paclitaxel in a TNBC model (EMT6, immune excluded with low TMB). This resulted in several complete responders compared to (chemo)immunotherapy alone, demonstrating the effectiveness of ablative UTMC treatment in cancer therapy [176]. Ablative UTMC was employed to explore immunogenic cell death in a TNBC model (4T1, immune excluded with low TMB); UTMC induced the translocation and expression of calreticulin and enhanced IL-12 and TNF-α, leading to increased infiltration of DC and CTL in both the tumor and TDLNs [177].

### 5.2. Non-Ablative FUS + ICI

HT has been tested for thermally-activated chemotherapeutic release, wherein priming with ICI therapy greatly enhances complete tumor regression [75]. This study combined TLR9 antagonist (CpG) and HT doxorubicin (Dox) encapsulation within liposomes and increased tumor antigen cross-presentation even at distant tumor sites. Complete tumor rejection was greatest in metastatic HER2^+^BC (NDL, immune-excluded TME with low TMB) when one week of aPD-1 priming was added to the HT + CpG + Dox protocol, which provoked systemic adaptive immunity. They reported enhanced Dox delivery and improved efficiency of combination therapy, with 90% of treated mice completely rejecting the tumor. The combined group with primed immunotherapy (CpG and aPD1) represented the highest level of TAA presentation and cross-presentation, which improved CTL infiltration in both treated and distant tumors [75].

Anti-tumor immune responses were more robust following mechanical perturbation compared with HT or T-HIFU, but the mechanism is yet to be discovered [6]. pFUS was used in a pancreatic murine model (KPC, immune-excluded TME with low TMB) to enhance the anti-cancer efficacy of aCTLA-4 and aPD-1, wherein the combined treatment extended mouse survival and increased CTL and DC infiltration [178]. Treatments combining UTMC and ICI have shown enhanced recruitment and activation of DCs (immediately), Tregs (transient, increased on day 1 but subsided by day 3), and CTLs (continuous increase starting from day 1 through day 18) [6]. Additionally, these treatments have been found to reduce the gene expression of anergy pathways, resulting in tumor growth suppression and prolonged survival in mice [118,161,166]. Several groups have combined UTMC with aPD-L1 to increase the delivery of aPD-L1 to brain tumors. Sheybani et al. (2021) reported that the timing of ICI injection is crucial for increasing its delivery [179]. In this study, administering aCD47—an immune checkpoint molecule on the surface of macrophages—***after*** UTMC treatment showed the highest aCD47 concentration with decreased tumor growth and increased mouse survival [179]. Li et al. (2021) demonstrated that UTMC, resulting in increased perfusion, improved the efficacy of aPDL1 treatment and increased infiltration of CTLs by 24%. Their treatment of CRC-MSI (MC38, immune excluded with high TMB) led to tumor vascular normalization and improved survival rates in the UTMC + aPDL1 group. The treatment also enhanced CTL activity, as evidenced by increased IFN-γ and granzyme B [90]. Therefore, normalizing tumor vascularization is proposed as an efficient way of either decreasing IFP or increasing TILs [90,148]. UTMC was compared with T-HIFU in a MC38 murine model in combination with aPD-L1 [180]. Although the authors reported ablative UTMC, we believe it should be considered non-ablative, given that tumor perfusion was maintained after UTMC. In this study, UTMC was more efficient in inducing an abscopal effect; mechanical perturbation by UTMC improved mouse survival following UTMC + aPD-L1 therapy by increasing the expression of DAMPs; enhancing the tumor infiltration of CTLs, DCs, TAMs; and reducing Tregs and MDSCs [180].

UTMC has also been used to target the spleen to modulate circulating immune cells instead of tumor-infiltrative ones. In a murine model of Lewis lung cancer (NSCLC, inflamed TME with high TMB), the splenic area was treated by UTMC to reduce CD71^+^ erythroid progenitor cells (CECs) [181]. CECs are immature red blood cells that contribute to immune regulation [182,183]. CECs inhibit CTLs, CD4^+^ T cell proliferation, and Th cell differentiation within the TME, mainly through the suppression of IFN-γ via TGF-β induction. Therefore, Tan et al. (2021) used UTMC to alleviate splenic CEC immunosuppression. Splenic CEC reduction increased splenic CTLs (MI = between 0.98 and 1.03). The combination of splenic UTMC with systemic aPD-L1 inhibited tumor growth and enhanced the frequency of splenic IFN-γ^+^ CTLs and IFN-γ^+^ CD4^+^ cells while reducing TGF-β^+^ CD11b^+^ cells [181].

Overall, it appears that mechanical perturbations (ablative or non-ablative) may be more efficient than T-HIFU in promoting anti-tumor immune responses. However, more comparative studies are needed to optimize and characterize how FUS modalities induce immunomodulation.

## 6. Translational Challenges and Outlook

Preclinical studies have provided evidence that FUS +/− MB can induce innate and adaptive immune activation, particularly when combined with ICI. Notably, several ultrasound devices are FDA approved, such as Sonablate, Ablatherm, Focal-One, Tulsa-pro^®^ [184], and Edison^TM^ [185] for HIFU. Additionally, Exablate, NaviFUS^®^, and Sonocloud1/9^®^ are typically used for UTMC to enhance drug delivery [186]. Sonalleve [187] and Sonotherm [188] are other FDA-approved devices used for HT. FDA-approved MBs include Sonovue^®^, Sonazoid^TM^, and Definity^®^ [189]. Currently, three clinical trials are underway to evaluate the safety and effectiveness of combined HIFU + aPD-1 therapy (NCT03237572 in metastatic breast cancers and NCT04116320 in advanced solid tumors) and UTMC + aPD-1 (NCT04021420 in melanoma brain metastases). These important trials are expected to help delineate the potential of FUS +/− MB in the clinical setting.

In this context, it seems crucial to continue investigating and understanding the mechanisms that make FUS and ICI synergize and to capitalize on these findings. Unraveling the intricate mechanisms of action of FUS +/− MB therapy, whether used as a standalone treatment or in combination with chemo-, radio-, or immunotherapy, will be crucial in bringing FUS +/− MB to the clinical arena. For instance, it is imperative to acknowledge that the immune system functions as a complex and interconnected system; while T cells play a crucial role as cytotoxic effector cells, it is essential to recognize that they do not operate in isolation. Rather, their function involves an intricate interplay among numerous mediators at both the cellular and molecular levels. For example, a recent study by Joiner et al. (2022) delineated the involvement of B cells in improving anti-tumor immune responses following a single treatment of UTMC in a pancreatic mouse model [190]. In this fashion, attention to the functionality of other kinds of immune cells and, in particular, different cytokines is indispensable to improve the efficiency of combined FUS + MB. This point may assist researchers in identifying the key contributors to immunity induced by FUS +/− MB treatment because, in some reports, despite the presence of partial or complete responders, no changes in the status of CTLs were observed [166,176].

It is likely that more personalized strategies in FUS + MB-mediated cancer therapy will be needed, guided by the identification of biomarkers stratifying the responders to FUS or to a precise type of FUS therapy. Furthermore, the cancer biology and cancer genetics should be considered to determine which kind of monotherapy or combined therapy (chemo-, immuno-, or radiotherapy) is suitable for applying to the right FUS modality, including the tumor type, patient, and timing. Combining several drugs is not always beneficial, as it may increase irAE for immunotherapies or severe toxicity in the case of chemotherapies [191]. Nowadays, the eligibility criteria for ICI therapy appear to be transitioning from the origin of tissue and the immunoscore to the molecular and cellular characterization of tumors. Personalized multi-omics and complete immune profiling could allow for the identification of signatures of candidate tumors before therapy. For example, the roles of other cell types beyond T cells (myeloid cells, fibroblasts) could be detrimental to the ability of FUS treatments, either alone or in combination with other therapeutics, to improve ICI efficacy. Understanding these interactions will enable scientists to determine if there is a suitable FUS modality for each tumor and patient, narrowing the gap toward personalized FUS immunotherapy.

While preclinical studies are encouraging, it is essential to recognize that animal physiology differs from human physiology. Furthermore, the intrinsic intra and inter-patient variability in cancer genetics can also influence treatment efficacy. In this review (see Figure 4), we aimed to categorize FUS studies among different preclinical tumor models that have been studied. The main objective was to categorize the TME immune profile. This will hopefully help in the identification of gaps and opportunities for future studies. However, several factors limit the applicability of these models to human tumors, and it is important to consider these factors when analyzing Figure 4 and Supplementary Table S1. Firstly, syngeneic tumors are not naturally occurring; instead, they are generated by implanting a specific MHC-matched cell line derived from fully developed tumors, typically through subcutaneous implantation. Secondly, the tumor cell lines used in syngeneic models originate from various tissue types or organs, which have distinct TMEs that differ from the site of subcutaneous implantation. Lastly, these implanted tumors progress at a much faster rate compared to spontaneously occurring tumors. These observations underscore the fact that syngeneic tumor models do not accurately reflect the typical progression of tumors in patients. In contrast, genetically engineered mouse (GEM) models of cancer, either germline or conditional, offer greater physiological relevance, as they replicate the appropriate kinetics and stepwise progression from tumor initiation to tumor establishment at the specific site of tumor origin [192]. This discrepancy in tumor initiation and growth location between subcutaneous syngeneic models and GEM models likely results in different inherent immune infiltration profiles and, consequently, diverse anti-tumor immune responses. For instance, spontaneous lung or pancreatic tumors in GEM models elicit weak T-cell responses within the tumor that diminish over time, whereas subcutaneously implanted tumors derived from cell lines of the same spontaneous tumors induce significantly greater T-cell infiltration and anti-tumor responses [193,194].

## 7. Conclusions

In closing, FUS +/− MB treatment is promising in various clinical disorders, including cancer, offering non-invasiveness, spatial targeting, and real-time imaging capabilities. In this review, we described the biological and immunological effects of FUS, allowing researchers to overcome immunological barriers, provoke anti-tumor immune responses, and improve the efficiency of cancer ICI therapy. Preclinical studies have been collected and categorized comprehensively based on their immune profiles. Our data collection indicates that melanoma and TNBC are the most extensively FUS studied models. CRC-MSI, HER2^+^ breast cancer, and glioma are the second-most studied. pFUS was exclusively studied in melanoma and TNBC tumor models, while UTMC was frequently applied to an immune-excluded TME with low TMB. Notably, T-HIFU was mostly studied in HER2^+^BC models so far and was not studied in other immune-excluded or immune desert models. Finally, our analysis also supports that various cancer types, especially immune desert tumor models, such as prostate and HR^+^ breast cancer, for example, have not yet been investigated using FUS, presenting an unexplored opportunity for future research in this growing, challenging, and promising field.

## Figures and Tables

**Table 1:** Differences in sonication parameters, physical mechanisms of action, and biological and immunological effects of FUS modalities.

		FUS Modality	Sonication Parameters	Physical Mechanisms and Effects	Biological Effects	Immune Effects
**Ablative**	**High Intensity FUS**	**T-HIFU** [62,63,64,65,66]	I_SPTA_ > 1000 W/cm^2^ P = 3–70 MPa F = 0.2–20 MHz DC% = 100%	-Temperature increase to ~60–85 °C -Stable and inertial cavitation	-Coagulative tissue necrosis -Increase TAAs -Increase DAMPs -Increase lymphatic drainage	-Inflammatory cytokines -Maturity of DCs -Increase TIL population -Heat-shock protein release
		**M-HIFU** [66,67,68,69,70]	-Histotripsy I_SPTA_ < 300 W/cm^2^ P = 20–80 MPa F = 0.2–3 MHz DC% ~0.01% -Boiling histotripsy I_SPTA_ < 600 W/cm^2^ P = 10–20 MPa F = 0.2–3 MHz DC% ~4%	-Mechanical histotripsy (inertial cavitation, micro-jetting, streaming, and shear stress) -Boiling histotripsy (MB boiling; thermal and mechanical effect, shockwaves)	-Coagulative tissue fractionation and liquefaction -TAA increase -Non-thermally damaged TAA release -Microhemorrhage	-Immunogenic cell death -Better APC activation than HT -TIL increase
**Non-ablative**		**HT** [66,71,72,73,74,75]	I_SPTA_ > 10 W/cm^2^ P = 0.1–5 MPa F = 0.2–3 MHz DC% = 0.5–100%	-Temperature increase to ~45 °C	-Blood perfusion increase -Tissue oxygenation improvement -Thermally controlled drug release -Protein and DNA damage-induced cell arrest and apoptosis - IFP reduction	-HSP70 increase -Increase in M1 macrophages and NK cells -DC activation -Increase in TNFα and IFNγ -Decrease in MDSCs and IL-10
	**Low Intensity FUS**	**pFUS** [76,77,78]	I_SPTA_ < 100 W/cm^2^ PNP = 0.1–10 MPa F < 3 MHz DC% = 0.5–20%	-Mostly non-thermal effects -Mild heating (<5 degrees)	-Mechanical forces without significant heating -Low cellular damage -Immunomodulation - DNA strand break -Calreticulin translocation	-Expression of CCTFs and CAM -Immune cell homing -Innate and adaptive immune response within the TME (NK cells, DCs, Th1 cells, and CTLs) -Increase in inflammatory immune cells in TDLNs and the spleen
		**UTMC** [67,79]	I_SPTA_ < 10 W/cm^2^ PNP = 0.1–4 MPa F = 0.2–3 MHz DC% = 0.5–50%	-Sonoporation -Shear stress	-Targeted drug release -Vascular permeabilization -BBB/BTB opening -Reduction in IFP -Reduction in hypoxia -ATP release	-Vascular inflammation -IL-2 and IFNγ increase -TILs -Expression of regulatory genes in MDSCs -Decreased expression of genes responsible for T-cell anergy (i.e., *Grail*, *Itch*, *Cbib*, *Grg4*)

***Acronyms***: I_SPTA_: spatial peak temporal average intensity; F: frequency; P: pressure; DC%: duty cycle (%); HIFU: high-intensity focused ultrasound; T-HIFU: thermal HIFU; M-HIFU: mechanical HIFU; pFUS: pulsed focused ultrasound; TDLN: tumor-draining lymph node; UTMC: ultrasound-targeted microbubble cavitation.

## Supplementary Materials

The following supporting information can be downloaded at: https://www.mdpi.com/article/10.3390/pharmaceutics16030411/s1, Table S1: Summary of bioeffects following targeted FUS with/without MB treatment.

## Abbreviations

Ado: adenosine; APC: antigen-presenting cell; ATP: adenosine triphosphate; BBB: blood–brain barrier; CRC: colorectal carcinoma; CTL: cytotoxic T cell; CTLA-4: cytotoxic T lymphocyte-associated protein 4; DAMP: danger-associated molecular pattern; DC: dendritic cell; DC%: duty cycle; eATP: extracellular adenosine triphosphate; ECM: extracellular matrix; FUS: focused ultrasound; HIFU: high-intensity focused ultrasound; HMGB1: high mobility group box 1; HSP: heat-shock protein; HT: hyperthermia; ICAM-1: intracellular adhesion molecule-1; ICI: immune checkpoint inhibitor; IDO: indoleamine 2,3-dioxygenase; IFP: interstitial fluid pressure; IL-10: interleukin-10; I_SPTA_: spatial peak temporal average intensity; MB: microbubble; MDSC: myeloid derived suppressor cell; M-HIFU: mechanical HIFU; MSI: microsatellite instable; MSS: microsatellite stable; NK: natural killer; NSCLC: non-small cell lung cancer; PD-1: programed cell death-1; PDL1: programmed cell death ligand-1; pFUS: pulsed FUS; PNP: peak negative pressure; TAA: tumor-associated antigen; TAM: tumor-associated macrophage; TDLN: tumor-draining lymph node; TGF-β: tumor growth factor β; Th: helper T cell; T-HIFU: thermal HIFU; TIL: tumor-infiltrating lymphocyte; TMB: tumor mutation burden; TME: tumor microenvironment; TNBC: triple-negative breast cancer; TNF-α: tumor necrosis factor α; Treg: regulatory T cell; UTMC: ultrasound-targeted microbubble cavitation; VEGF: vascular endothelial growth factor.

## References

[1] Sonpavde G.P., Grivas P., Lin Y., Hennessy D., Hunt J.D. (2021). Immune-related adverse events with pd-1 versus pd-l1 inhibitors: A meta-analysis of 8730 patients from clinical trials. Future Oncol..

[2] du Rusquec P., de Calbiac O., Robert M., Campone M., Frenel J.S. (2019). Clinical utility of pembrolizumab in the management of advanced solid tumors: An evidence-based review on the emerging new data. Cancer Manag. Res..

[3] O’Brien M., Paz-Ares L., Marreaud S., Dafni U., Oselin K., Havel L., Esteban E., Isla D., Martinez-Marti A., Faehling M. (2022). Pembrolizumab versus placebo as adjuvant therapy for completely resected stage ib–iiia non-small-cell lung cancer (pearls/keynote-091): An interim analysis of a randomised, triple-blind, phase 3 trial. Lancet Oncol..

[4] Burtness B., Harrington K., Greil R., Soulières D., Tahara M., De Castro G., Psyrri A., Rotllan N.B., Neupane P., Bratland Å. (2018). Keynote-048: Phase III study of first-line pembrolizumab (P) for recurrent/metastatic head and neck squamous cell carcinoma (r/m hnscc). Ann. Oncol..

[5] Choueiri T.K., Quinn D.I., Zhang T., Gurney H., Doshi G.K., Cobb P.W., Parnis F., Lee J.-L., Park S.H., Semenov A. (2018). keynote-564: A phase 3, randomized, double blind, trial of pembrolizumab in the adjuvant treatment of renal cell carcinoma. J. Clin. Oncol..

[6] Joiner J.B., Pylayeva-Gupta Y., Dayton P.A. (2020). Focused Ultrasound for Immunomodulation of the Tumor Microenvironment. J. Immunol..

[7] Meng Y., Pople C.B., Lea-Banks H., Abrahao A., Davidson B., Suppiah S., Vecchio L.M., Samuel N., Mahmud F., Hynynen K. (2019). Safety and efficacy of focused ultrasound induced blood-brain barrier opening, an integrative review of animal and human studies. J. Control Release.

[8] Hu Z., Yang X.Y., Liu Y., Sankin G.N., Pua E.C., Morse M.A., Lyerly H.K., Clay T.M., Zhong P. (2007). Investigation of HIFU-induced anti-tumor immunity in a murine tumor model. J. Transl. Med..

[9] Snipstad S., Vikedal K., Maardalen M., Kurbatskaya A., Sulheim E., Davies C.L. (2021). Ultrasound and microbubbles to beat barriers in tumors: Improving delivery of nanomedicine. Adv. Drug Deliv. Rev..

[10] Jung O., Thomas A., Burks S.R., Dustin M.L., Frank J.A., Ferrer M., Stride E. (2022). Neuroinflammation associated with ultrasound-mediated permeabilization of the blood-brain barrier. Trends Neurosci..

[11] Liu Y.-T., Sun Z.-J. (2021). Turning cold tumors into hot tumors by improving T-cell infiltration. Theranostics.

[12] O’Donnell J.S., Teng M.W.L., Smyth M.J. (2019). Cancer immunoediting and resistance to T cell-based immunotherapy. Nat. Rev. Clin. Oncol..

[13] Gubin M.M., Vesely M.D. (2022). Cancer Immunoediting in the Era of Immuno-oncology. Clin. Cancer Res..

[14] Varade J., Magadan S., Gonzalez-Fernandez A. (2021). Human immunology and immunotherapy: Main achievements and challenges. Cell Mol. Immunol..

[15] Altmann D.M. (2018). A Nobel Prize-worthy pursuit: Cancer immunology and harnessing immunity to tumour neoantigens. Immunology.

[16] Sharpe A.H., Pauken K.E. (2018). The diverse functions of the PD1 inhibitory pathway. Nat. Rev. Immunol..

[17] Carlino M.S., Larkin J., Long G.V. (2021). Immune checkpoint inhibitors in melanoma. Lancet.

[18] Adams S., Loi S., Toppmeyer D., Cescon D.W., De Laurentiis M., Nanda R., Winer E.P., Mukai H., Tamura K., Armstrong A. (2019). Pembrolizumab monotherapy for previously untreated, PD-L1-positive, metastatic triple-negative breast cancer: Cohort B of the phase ii keynote-086 study. Ann. Oncol..

[19] Hui R., Gandhi L., Carcereny Costa E., Felip E., Ahn M.-J., Eder J.P., Balmanoukian A.S., Leighl N.B., Aggarwal C., Horn L. (2016). Long-term OS for patients with advanced NSCLC enrolled in the keynote-001 study of pembrolizumab (pembro). J. Clin. Oncol..

[20] Garon E.B., Rizvi N.A., Hui R., Leighl N., Balmanoukian A.S., Eder J.P., Patnaik A., Aggarwal C., Gubens M., Horn L. (2015). Pembrolizumab for the treatment of non-small-cell lung cancer. N. Engl. J. Med..

[21] Powles T., Csőszi T., Özgüroğlu M., Matsubara N., Géczi L., Cheng S.Y., Fradet Y., Oudard S., Vulsteke C., Barrera R.M. (2021). Pembrolizumab alone or combined with chemotherapy versus chemotherapy as first-line therapy for advanced urothelial carcinoma (keynote-361): A randomised, open-label, phase 3 trial. Lancet Oncol..

[22] Colombo N., Dubot C., Lorusso D., Caceres M.V., Hasegawa K., Shapira-Frommer R., Tewari K.S., Salman P., Hoyos Usta E., Yanez E. (2021). Pembrolizumab for Persistent, Recurrent, or Metastatic Cervical Cancer. N. Engl. J. Med..

[23] Ardolino L., Joshua A. (2019). Immune checkpoint inhibitors in malignancy. Aust. Prescr..

[24] Marin-Acevedo J.A., Kimbrough E.O., Lou Y. (2021). Next generation of immune checkpoint inhibitors and beyond. J. Hematol. Oncol..

[25] Allard B., Allard D., Buisseret L., Stagg J. (2020). The adenosine pathway in immuno-oncology. Nat. Rev. Clin. Oncol..

[26] Allard B., Longhi M.S., Robson S.C., Stagg J. (2017). The ectonucleotidases CD 39 and CD 73: Novel checkpoint inhibitor targets. Immunol. Rev..

[27] Linnemann C., Schildberg F.A., Schurich A., Diehl L., Hegenbarth S.I., Endl E., Lacher S., Müller C.E., Frey J., Simeoni L. (2009). Adenosine regulates cd8 t-cell priming by inhibition of membrane-proximal t-cell receptor signalling. Immunology.

[28] Zhang H., Conrad D.M., Butler J.J., Zhao C., Blay J., Hoskin D.W. (2004). Adenosine acts through a2 receptors to inhibit il-2-induced tyrosine phosphorylation of stat5 in T lymphocytes: Role of cyclic adenosine 3′, 5′-monophosphate and phosphatases. J. Immunol..

[29] Sorrentino C., Hossain F., Rodriguez P.C., Sierra R.A., Pannuti A., Hatfield S., Osborne B.A., Minter L.M., Miele L., Morello S. (2019). Adenosine A2A receptor stimulation inhibits tcr-induced Notch1 activation in cd8+ t-cells. Front. Immunol..

[30] Romio M., Reinbeck B., Bongardt S., Hüls S., Burghoff S., Schrader J. (2011). Extracellular purine metabolism and signaling of cd73-derived adenosine in murine Treg and Teff cells. Am. J. Physiol. Cell Physiol..

[31] Leone R.D., Sun I.-M., Oh M.-H., Sun I.-H., Wen J., Englert J., Powell J.D. (2018). Inhibition of the adenosine A2a receptor modulates expression of T cell coinhibitory receptors and improves effector function for enhanced checkpoint blockade and act in murine cancer models. Cancer Immunol. Immunother..

[32] Ohta A., Kini R., Ohta A., Subramanian M., Madasu M., Sitkovsky M. (2012). The development and immunosuppressive functions of cd4+ cd25+ foxp3+ regulatory T cells are under influence of the adenosine-a2a adenosine receptor pathway. Front. Immunol..

[33] Zarek P.E., Huang C.-T., Lutz E.R., Kowalski J., Horton M.R., Linden J., Drake C.G., Powell J.D. (2008). A2a receptor signaling promotes peripheral tolerance by inducing T-cell anergy and the generation of adaptive regulatory T cells. Blood.

[34] Jarnicki A.G., Lysaght J., Todryk S., Mills K.H. (2006). Suppression of antitumor immunity by il-10 and tgf-β-producing T cells infiltrating the growing tumor: Influence of tumor environment on the induction of CD4+ and CD8+ regulatory T cells. J. Immun..

[35] Wrzesinski S.H., Wan Y.Y., Flavell R.A. (2007). Transforming growth factor-β and the immune response: Implications for anticancer therapy. Clin. Cancer Res..

[36] Morris R.M., Mortimer T.O., O’Neill K.L. (2022). Cytokines: Can Cancer Get the Message?. Cancers.

[37] Hatfield S.M., Kjaergaard J., Lukashev D., Schreiber T.H., Belikoff B., Abbott R., Sethumadhavan S., Philbrook P., Ko K., Cannici R. (2015). Immunological mechanisms of the antitumor effects of supplemental oxygenation. Sci. Transl. Med..

[38] Oft M. (2014). IL-10: Master switch from tumor-promoting inflammation to antitumor immunity. Cancer Immunol. Res..

[39] Akdis C.A., Blaser K. (2001). Mechanisms of interleukin-10-mediated immune suppression. Immunology.

[40] Neven B., Mamessier E., Bruneau J., Kaltenbach S., Kotlarz D., Suarez F., Masliah-Planchon J., Billot K., Canioni D., Frange P. (2013). A Mendelian predisposition to B-cell lymphoma caused by il-10r deficiency. Blood.

[41] Bai X., Yi M., Jiao Y., Chu Q., Wu K. (2019). Blocking TGF-β signaling to enhance the efficacy of immune checkpoint inhibitor. OncoTargets Ther..

[42] David C.J., Massagué J. (2018). Contextual determinants of TGFβ action in development, immunity and cancer. Nat. Rev. Mol. Cell Biol..

[43] Hegde P.S., Chen D.S. (2020). Top 10 Challenges in Cancer Immunotherapy. Immunity.

[44] McGranahan N., Furness A.J., Rosenthal R., Ramskov S., Lyngaa R., Saini S.K., Jamal-Hanjani M., Wilson G.A., Birkbak N.J., Hiley C.T. (2016). Clonal neoantigens elicit T cell immunoreactivity and sensitivity to immune checkpoint blockade. Science.

[45] Lower S.S., McGurk M.P., Clark A.G., Barbash D.A. (2018). Satellite DNA evolution: Old ideas, new approaches. Curr. Opin. Genet. Dev..

[46] Picard E., Verschoor C.P., Ma G.W., Pawelec G. (2020). Relationships between immune landscapes, genetic subtypes and responses to immunotherapy in colorectal cancer. Front. Immunol..

[47] Addeo A., Friedlaender A., Banna G.L., Weiss G.J. (2021). TMB or not tmb as a biomarker: That is the question. Crit. Rev. Oncol. Hematol..

[48] Choucair K., Morand S., Stanbery L., Edelman G., Dworkin L., Nemunaitis J. (2020). TMB: A promising immune-response biomarker, and potential spearhead in advancing targeted therapy trials. Cancer Gene Ther..

[49] Rosenberg J.E., Hoffman-Censits J., Powles T., Van Der Heijden M.S., Balar A.V., Necchi A., Dawson N., O’Donnell P.H., Balmanoukian A., Loriot Y. (2016). Atezolizumab in patients with locally advanced and metastatic urothelial carcinoma who have progressed following treatment with platinum-based chemotherapy: A single-arm, multicentre, phase 2 trial. Lancet.

[50] Rizvi N.A., Hellmann M.D., Snyder A., Kvistborg P., Makarov V., Havel J.J., Lee W., Yuan J., Wong P., Ho T.S. (2015). Cancer immunology. Mutational landscape determines sensitivity to PD-1 blockade in non-small cell lung cancer. Science.

[51] Cristescu R., Mogg R., Ayers M., Albright A., Murphy E., Yearley J., Sher X., Liu X.Q., Lu H., Nebozhyn M. (2018). Pan-tumor genomic biomarkers for PD-1 checkpoint blockade-based immunotherapy. Science.

[52] Samstein R.M., Lee C.H., Shoushtari A.N., Hellmann M.D., Shen R., Janjigian Y.Y., Barron D.A., Zehir A., Jordan E.J., Omuro A. (2019). Tumor mutational load predicts survival after immunotherapy across multiple cancer types. Nat. Genet..

[53] Galon J., Mlecnik B., Bindea G., Angell H.K., Berger A., Lagorce C., Lugli A., Zlobec I., Hartmann A., Bifulco C. (2014). Towards the introduction of the ‘Immunoscore’ in the classification of malignant tumours. J. Pathol..

[54] Galon J., Bruni D. (2019). Approaches to treat immune hot, altered and cold tumours with combination immunotherapies. Nat. Rev. Drug Discov..

[55] Jenkins R.W., Barbie D.A., Flaherty K.T. (2018). Mechanisms of resistance to immune checkpoint inhibitors. Br. J. Cancer.

[56] Joshi K., de Massy M.R., Ismail M., Reading J.L., Uddin I., Woolston A., Hatipoglu E., Oakes T., Rosenthal R., Peacock T. (2019). Spatial heterogeneity of the T cell receptor repertoire reflects the mutational landscape in lung cancer. Nat. Med..

[57] Sugiura A., Rathmell J.C. (2018). Metabolic Barriers to T Cell Function in Tumors. J. Immunol..

[58] Mariathasan S., Turley S., Nickles D., Castiglioni A., Yuen K., Wang Y., Kadel E. (2018). TGFβ attenuates tumour response to pd-l1 blockade by contributing to exclusion of T cells. Nature. Nat..

[59] Galluzzi L., Spranger S., Fuchs E., Lopez-Soto A. (2019). WNT Signaling in Cancer Immunosurveillance. Trends Cell Biol..

[60] Izadifar Z., Izadifar Z., Chapman D., Babyn P. (2020). An Introduction to High Intensity Focused Ultrasound: Systematic Review on Principles, Devices, and Clinical Applications. J. Clin. Med..

[61] Shi G., Zhong M., Ye F., Zhang X. (2019). Low-frequency HIFU induced cancer immunotherapy: Tempting challenges and potential opportunities. Cancer Biol. Med..

[62] Bove T., Zawada T., Serup J., Jessen A., Poli M. (2019). High-frequency (20-mhz) high-intensity focused ultrasound (hifu) system for dermal intervention: Preclinical evaluation in skin equivalents. Skin Res. Technol..

[63] Zhao L.Y., Zou J.Z., Chen Z.G., Liu S., Jiao J., Wu F. (2016). Acoustic Cavitation Enhances Focused Ultrasound Ablation with Phase-Shift Inorganic Perfluorohexane Nanoemulsions: An In Vitro Study Using a Clinical Device. Biomed. Res. Int..

[64] Zhou Y., Wong C.O., Cho K.J., van der Hoeven D., Liang H., Thakur D.P., Luo J., Babic M., Zinsmaier K.E., Zhu M.X. (2015). SIGNAL TRANSDUCTION. Membrane potential modulates plasma membrane phospholipid dynamics and K-Ras signaling. Science.

[65] Wan M., Feng Y., Ter Haar G. (2015). Cavitation in Biomedicine.

[66] Kim C., Lim M., Woodworth G.F., Arvanitis C.D. (2022). The roles of thermal and mechanical stress in focused ultrasound-mediated immunomodulation and immunotherapy for central nervous system tumors. J. Neurooncol..

[67] Izadifar Z., Babyn P., Chapman D. (2017). Mechanical and biological effects of ultrasound: A review of present knowledge. Ultrasound Med. Biol..

[68] Riesberg G., Bigelow T.A., Stessman D.J., Spalding M.H., Yao L., Wang T., Xu J. (2014). Flow rate and duty cycle effects in lysis of Chlamydomonas reinhardtii using high-energy pulsed focused ultrasound. J. Acoust. Soc. Am..

[69] Holt R.G., Roy R.A. (2001). Measurements of bubble-enhanced heating from focused, mhz-frequency ultrasound in a tissue-mimicking material. Ultrasound Med. Biol..

[70] Xu Z., Hall T.L., Vlaisavljevich E., Lee F.T. (2021). Histotripsy: The first noninvasive, non-ionizing, non-thermal ablation technique based on ultrasound. Int. J. Hyperth..

[71] Guillemin P.C., Gui L., Lorton O., Zilli T., Crowe L.A., Desgranges S., Montet X., Terraz S., Miralbell R., Salomir R. (2019). Mild hyperthermia by mr-guided focused ultrasound in an ex vivo model of osteolytic bone tumour: Optimization of the spatio-temporal control of the delivered temperature. J. Transl. Med..

[72] Speed C. (2001). Therapeutic ultrasound in soft tissue lesions. Rheumatology.

[73] Partanen A., Yarmolenko P.S., Viitala A., Appanaboyina S., Haemmerich D., Ranjan A., Jacobs G., Woods D., Enholm J., Wood B.J. (2012). Mild hyperthermia with magnetic resonance-guided high-intensity focused ultrasound for applications in drug delivery. Int. J. Hyperth..

[74] Frazier N., Payne A., de Bever J., Dillon C., Panda A., Subrahmanyam N., Ghandehari H. (2016). High intensity focused ultrasound hyperthermia for enhanced macromolecular delivery. J. Control Release.

[75] Kheirolomoom A., Silvestrini M.T., Ingham E.S., Mahakian L.M., Tam S.M., Tumbale S.K., Foiret J., Hubbard N.E., Borowsky A.D., Ferrara K.W. (2019). Combining activatable nanodelivery with immunotherapy in a murine breast cancer model. J. Control Release.

[76] Aydin O., Chandran P., Lorsung R.R., Cohen G., Burks S.R., Frank J.A. (2019). The Proteomic Effects of Pulsed Focused Ultrasound on Tumor Microenvironments of Murine Melanoma and Breast Cancer Models. Ultrasound Med. Biol..

[77] Cohen G., Chandran P., Lorsung R.M., Aydin O., Tomlinson L.E., Rosenblatt R.B., Burks S.R., Frank J.A. (2021). Pulsed-Focused Ultrasound Slows B16 Melanoma and 4T1 Breast Tumor Growth through Differential Tumor Microenvironmental Changes. Cancers.

[78] Mohammadabadi A., Huynh R.N., Wadajkar A.S., Lapidus R.G., Kim A.J., Raub C.B., Frenkel V. (2020). Pulsed focused ultrasound lowers interstitial fluid pressure and increases nanoparticle delivery and penetration in head and neck squamous cell carcinoma xenograft tumors. Phys. Med. Biol..

[79] Michon S., Rodier F., Yu F.T.H. (2022). Targeted Anti-Cancer Provascular Therapy Using Ultrasound, Microbubbles, and Nitrite to Increase Radiotherapy Efficacy. Bioconjug. Chem..

[80] Elhelf I.A.S., Albahar H., Shah U., Oto A., Cressman E., Almekkawy M. (2018). High intensity focused ultrasound: The fundamentals, clinical applications and research trends. Diagn. Interv. Imaging.

[81] Ho Y.J., Li J.P., Fan C.H., Liu H.L., Yeh C.K. (2020). Ultrasound in tumor immunotherapy: Current status and future developments. J. Control Release.

[82] Xia J.Z., Xie F.L., Ran L.F., Xie X.P., Fan Y.M., Wu F. (2012). High-intensity focused ultrasound tumor ablation activates autologous tumor-specific cytotoxic T lymphocytes. Ultrasound Med. Biol..

[83] Deng J., Zhang Y., Feng J., Wu F. (2010). Dendritic cells loaded with ultrasound-ablated tumour induce in vivo specific antitumour immune responses. Ultrasound Med. Biol..

[84] Fite B.Z., Wang J., Kare A.J., Ilovitsh A., Chavez M., Ilovitsh T., Zhang N., Chen W., Robinson E., Zhang H. (2021). Immune modulation resulting from mr-guided high intensity focused ultrasound in a model of murine breast cancer. Sci. Rep..

[85] Silvestrini M.T., Ingham E.S., Mahakian L.M., Kheirolomoom A., Liu Y., Fite B.Z., Tam S.M., Tucci S.T., Watson K.D., Wong A.W. (2017). Priming is key to effective incorporation of image-guided thermal ablation into immunotherapy protocols. JCI Insight.

[86] Lundt J.E., Allen S.P., Shi J., Hall T.L., Cain C.A., Xu Z. (2017). Non-invasive, Rapid Ablation of Tissue Volume Using Histotripsy. Ultrasound Med. Biol..

[87] Izadifar Z., Babyn P., Chapman D. (2019). Ultrasound cavitation/microbubble detection and medical applications. J. Med. Biol. Eng..

[88] Khokhlova V.A., Fowlkes J.B., Roberts W.W., Schade G.R., Xu Z., Khokhlova T.D., Hall T.L., Maxwell A.D., Wang Y.N., Cain C.A. (2015). Histotripsy methods in mechanical disintegration of tissue: Towards clinical applications. Int. J. Hyperth..

[89] Vlaisavljevich E., Kim Y., Owens G., Roberts W., Cain C., Xu Z. (2014). Effects of tissue mechanical properties on susceptibility to histotripsy-induced tissue damage. Phys. Med. Biol..

[90] Li N., Tang J., Yang J., Zhu B., Wang X., Luo Y., Yang H., Jang F., Zou J., Liu Z. (2021). Tumor perfusion enhancement by ultrasound stimulated microbubbles potentiates PD-L1 blockade of MC38 colon cancer in mice. Cancer Lett..

[91] Korbelik M., Banath J., Saw K.M., Zhang W., Čiplys E. (2015). Calreticulin as cancer treatment adjuvant: Combination with photodynamic therapy and photodynamic therapy-generated vaccines. Front. Oncol..

[92] Qu S., Worlikar T., Felsted A.E., Ganguly A., Beems M.V., Hubbard R., Pepple A.L., Kevelin A.A., Garavaglia H., Dib J. (2020). Non-thermal histotripsy tumor ablation promotes abscopal immune responses that enhance cancer immunotherapy. J. Immunother. Cancer.

[93] Pahk K.J., Shin C.H., Bae I.Y., Yang Y., Kim S.H., Pahk K., Kim H., Oh S.J. (2019). Boiling Histotripsy-induced Partial Mechanical Ablation Modulates Tumour Microenvironment by Promoting Immunogenic Cell Death of Cancers. Sci. Rep..

[94] Pisetsky D. (2011). Cell death in the pathogenesis of immune-mediated diseases: The role of HMGB1 and DAMP-PAMP complexes. Swiss Med. Wkly..

[95] Iwanicki I., Wu L.L., Flores-Guzman F., Sundland R., Viza-Gomes P., Nordgren R., Centner C.S., Kandel J.J., Applebaum M.A., Bader K.B. (2023). Histotripsy induces apoptosis and reduces hypoxia in a neuroblastoma xenograft model. Int. J. Hyperth..

[96] Eranki A., Srinivasan P., Ries M., Kim A., Lazarski C.A., Rossi C.T., Khokhlova T.D., Wilson E., Knoblach S.M., Sharma K.V. (2020). High-Intensity Focused Ultrasound (HIFU) Triggers Immune Sensitization of Refractory Murine Neuroblastoma to Checkpoint Inhibitor TherapyHIFU with Immunotherapy Cure Refractory Murine Neuroblastoma. Clin. Cancer Res..

[97] Schade G.R., Wang Y.N., D’Andrea S., Hwang J.H., Liles W.C., Khokhlova T.D. (2019). Boiling Histotripsy Ablation of Renal Cell Carcinoma in the Eker Rat Promotes a Systemic Inflammatory Response. Ultrasound Med. Biol..

[98] Pepple A.L., Guy J.L., McGinnis R., Felsted A.E., Song B., Hubbard R., Worlikar T., Garavaglia H., Dib J., Chao H. (2023). Spatiotemporal local and abscopal cell death and immune responses to histotripsy focused ultrasound tumor ablation. Front. Immunol..

[99] Hoogenboom M., Eikelenboom D., den Brok M.H., Veltien A., Wassink M., Wesseling P., Dumont E., Futterer J.J., Adema G.J., Heerschap A. (2016). In vivo MR guided boiling histotripsy in a mouse tumor model evaluated by MRI and histopathology. NMR Biomed..

[100] Skandalakis G.P., Rivera D.R., Rizea C.D., Bouras A., Jesu Raj J.G., Bozec D., Hadjipanayis C.G. (2020). Hyperthermia treatment advances for brain tumors. Int. J. Hyperth..

[101] Suzuki R., Namai E., Oda Y., Nishiie N., Otake S., Koshima R., Hirata K., Taira Y., Utoguchi N., Negishi Y. (2010). Cancer gene therapy by il-12 gene delivery using liposomal bubbles and tumoral ultrasound exposure. J. Control Release.

[102] Huang S.H., Yang K.J., Wu J.C., Chang K.J., Wang S.M. (1999). Effects of hyperthermia on the cytoskeleton and focal adhesion proteins in a human thyroid carcinoma cell line. J. Cell Biochem..

[103] Oei A.L., Vriend L.E., Crezee J., Franken N.A., Krawczyk P.M. (2015). Effects of hyperthermia on DNA repair pathways: One treatment to inhibit them all. Radiat. Oncol..

[104] Ostberg J.R., Repasky E.A. (2006). Emerging evidence indicates that physiologically relevant thermal stress regulates dendritic cell function. Cancer Immunol. Immunother..

[105] Gouarderes S., Mingotaud A.-F., Vicendo P., Gibot L. (2020). Vascular and extracellular matrix remodeling by physical approaches to improve drug delivery at the tumor site. Expert. Opin. Drug Deliv..

[106] Chen Q., Hu Q., Dukhovlinova E., Chen G., Ahn S., Wang C., Ogunnaike E.A., Ligler F.S., Dotti G., Gu Z. (2019). Photothermal Therapy Promotes Tumor Infiltration and Antitumor Activity of car t Cells. Adv. Mater..

[107] Chang M., Hou Z., Wang M., Li C., Lin J. (2021). Recent Advances in Hyperthermia Therapy-Based Synergistic Immunotherapy. Adv. Mater..

[108] Ando K., Suzuki Y., Kaminuma T., Yoshimoto Y., Oike T., Okonogi N., Sato H., Tamaki T., Noda S.-E., Mimura K. (2018). Tumor-specific CD8-positive T cell-mediated antitumor immunity is implicated in the antitumor effect of local hyperthermia. Int. J. Hyperth..

[109] Dayanc B.E., Beachy S.H., Ostberg J.R., Repasky E.A. (2008). Dissecting the role of hyperthermia in natural killer cell mediated anti-tumor responses. Int. J. Hyperth..

[110] Chen Q., Appenheimer M.M., Muhitch J.B., Fisher D.T., Clancy K.A., Miecznikowski J.C., Wang W.C., Evans S.S. (2009). Thermal facilitation of lymphocyte trafficking involves temporal induction of intravascular ICAM-1. Microcirculation.

[111] Evans S.S., Repasky E.A., Fisher D.T. (2015). Fever and the thermal regulation of immunity: The immune system feels the heat. Nat. Rev. Immunol..

[112] Sheybani N.D., Witter A.R., Thim E.A., Yagita H., Bullock T.N.J., Price R.J. (2020). Combination of thermally ablative focused ultrasound with gemcitabine controls breast cancer via adaptive immunity. J. Immunother. Cancer.

[113] Han X., Wang R., Xu J., Chen Q., Liang C., Chen J., Zhao J., Chu J., Fan Q., Archibong E. (2019). In situ thermal ablation of tumors in combination with nano-adjuvant and immune checkpoint blockade to inhibit cancer metastasis and recurrence. Biomaterials.

[114] Burks S.R., Ziadloo A., Hancock H.A., Chaudhry A., Dean D.D., Lewis B.K., Frenkel V., Frank J.A. (2011). Investigation of cellular and molecular responses to pulsed focused ultrasound in a mouse model. PLoS ONE.

[115] Li T., Wang Y.N., Khokhlova T.D., D’Andrea S., Starr F., Chen H., McCune J.S., Risler L.J., Mashadi-Hossein A., Hingorani S.R. (2015). Pulsed High-Intensity Focused Ultrasound Enhances Delivery of Doxorubicin in a Preclinical Model of Pancreatic Cancer. Cancer Res..

[116] Lee S., Han H., Koo H., Na J.H., Yoon H.Y., Lee K.E., Lee H., Kim H., Kwon I.C., Kim K. (2017). Extracellular matrix remodeling in vivo for enhancing tumor-targeting efficiency of nanoparticle drug carriers using the pulsed high intensity focused ultrasound. J. Control Release.

[117] Hayashi F., Shigemura K., Maeda K., Hiraoka A., Maeshige N., Ooya T., Sung S.Y., Yang Y.M., Fujisawa M. (2022). Combined Treatment with Ultrasound and Immune Checkpoint Inhibitors for Prostate Cancer. J. Clin. Med..

[118] Bandyopadhyay S., Quinn T.J., Scandiuzzi L., Basu I., Partanen A., Tome W.A., Macian F., Guha C. (2016). Low-Intensity Focused Ultrasound Induces Reversal of Tumor-Induced t Cell Tolerance and Prevents Immune Escape. J. Immunol..

[119] Sirsi S.R., Borden M.A. (2012). Advances in ultrasound mediated gene therapy using microbubble contrast agents. Theranostics.

[120] Frinking P., Segers T., Luan Y., Tranquart F. (2020). Three Decades of Ultrasound Contrast Agents: A Review of the Past, Present and Future Improvements. Ultrasound Med. Biol..

[121] Chowdhury S.M., Abou-Elkacem L., Lee T., Dahl J., Lutz A.M. (2020). Ultrasound and microbubble mediated therapeutic delivery: Underlying mechanisms and future outlook. J. Control Release.

[122] Tran T.A., Le Guennec J.Y., Bougnoux P., Tranquart F., Bouakaz A. (2008). Characterization of cell membrane response to ultrasound activated microbubbles. IEEE Trans. Ultrason. Ferroelectr. Freq. Control.

[123] Kooiman K., van der Steen A.F., de Jong N. (2013). Role of intracellular calcium and reactive oxygen species in microbubble-mediated alterations of endothelial layer permeability. IEEE Trans. Ultrason. Ferroelectr. Freq. Control.

[124] Beekers I., Mastik F., Beurskens R., Tang P.Y., Vegter M., van der Steen A.F.W., de Jong N., Verweij M.D., Kooiman K. (2020). High-Resolution Imaging of Intracellular Calcium Fluctuations Caused by Oscillating Microbubbles. Ultrasound Med. Biol..

[125] Belcik J.T., Davidson B.P., Xie A., Wu M.D., Yadava M., Qi Y., Liang S., Chon C.R., Ammi A.Y., Field J. (2017). Augmentation of muscle blood flow by ultrasound cavitation is mediated by ATP and purinergic signaling. Circulation.

[126] Grygorczyk R., Boudreault F., Ponomarchuk O., Tan J.J., Furuya K., Goldgewicht J., Kenfack F.D., Yu F. (2021). Lytic Release of Cellular atp: Physiological Relevance and Therapeutic Applications. Life.

[127] Haugse R., Langer A., Murvold E.T., Costea D.E., Gjertsen B.T., Gilja O.H., Kotopoulis S., Ruiz de Garibay G., McCormack E. (2020). Low-intensity sonoporation-induced intracellular signalling of pancreatic cancer cells, fibroblasts and endothelial cells. Pharmaceutics.

[128] Liu X., Wang B., Ding H., Shi H., Liu J., Sun H. (2018). Low-intensity pulsed ultrasound in combination with SonoVue induces cytotoxicity of human renal glomerular endothelial cells via repression of the erk1/2 signaling pathway. Ren. Fail..

[129] Saha S., Bhanja P., Partanen A., Zhang W., Liu L., Tomé W., Guha C. (2014). Low intensity focused ultrasound (lofu) modulates unfolded protein response and sensitizes prostate cancer to 17aag. Oncoscience.

[130] Amate M., Goldgewicht J., Sellamuthu B., Stagg J., Yu F.T.H. (2020). The effect of ultrasound pulse length on microbubble cavitation induced antibody accumulation and distribution in a mouse model of breast cancer. Nanotheranostics.

[131] Frenkel V. (2008). Ultrasound mediated delivery of drugs and genes to solid tumors. Adv. Drug Deliv. Rev..

[132] Yang C., Du M., Yan F., Chen Z. (2019). Focused ultrasound improves NK-92MI cells infiltration into tumors. Front. Pharmacol..

[133] Qin J., Wang T.-Y., Willmann J.K., Escoffre J.-M., Bouakaz A. (2016). Sonoporation: Applications for Cancer Therapy. Therapeutic Ultrasound.

[134] Beekers I., Vegter M., Lattwein K.R., Mastik F., Beurskens R., van der Steen A.F., de Jong N., Verweij M.D., Kooiman K. (2020). Opening of endothelial cell–cell contacts due to sonoporation. J. Control Release.

[135] Heath C.H., Sorace A., Knowles J., Rosenthal E., Hoyt K. (2012). Microbubble therapy enhances anti-tumor properties of cisplatin and cetuximab in vitro and in vivo. Otolaryngol. Head. Neck Surg..

[136] Zderic V., Clark J.I., Vaezy S. (2004). Drug delivery into the eye with the use of ultrasound. J. Ultrasound Med..

[137] Schoellhammer C.M., Schroeder A., Maa R., Lauwers G.Y., Swiston A., Zervas M., Barman R., DiCiccio A.M., Brugge W.R., Anderson D.G. (2015). Ultrasound-mediated gastrointestinal drug delivery. Sci. Transl. Med..

[138] Azagury A., Khoury L., Enden G., Kost J. (2014). Ultrasound mediated transdermal drug delivery. Adv. Drug Deliv. Rev..

[139] Chen P.Y., Hsieh H.Y., Huang C.Y., Lin C.Y., Wei K.C., Liu H.L. (2015). Focused ultrasound-induced blood-brain barrier opening to enhance interleukin-12 delivery for brain tumor immunotherapy: A preclinical feasibility study. J. Transl. Med..

[140] Janowicz P.W., Leinenga G., Gotz J., Nisbet R.M. (2019). Ultrasound-mediated blood-brain barrier opening enhances delivery of therapeutically relevant formats of a tau-specific antibody. Sci. Rep..

[141] Zhang H., Sierra C., Kwon N., Karakatsani M.E., Jackson-Lewis V.R., Przedborski S., Konofagou E. Focused-Ultrasound Mediated Anti-Alpha-Synuclein Antibody Delivery for the Treatment of Parkinson’s Disease. Proceedings of the 2018 IEEE International Ultrasonics Symposium.

[142] Kovacs Z.I., Kim S., Jikaria N., Qureshi F., Milo B., Lewis B.K., Bresler M., Burks S.R., Frank J.A. (2017). Disrupting the blood-brain barrier by focused ultrasound induces sterile inflammation. Proc. Natl. Acad. Sci. USA.

[143] McMahon D., Hynynen K. (2017). Acute inflammatory response following increased blood-brain barrier permeability induced by focused ultrasound is dependent on microbubble dose. Theranostics.

[144] Sabbagh A., Beccaria K., Ling X., Marisetty A., Ott M., Caruso H., Barton E., Kong L.Y., Fang D., Latha K. (2021). Opening of the Blood-Brain Barrier Using Low-Intensity Pulsed Ultrasound Enhances Responses to Immunotherapy in Preclinical Glioma Models. Clin. Cancer Res..

[145] Curley C.T., Mead B.P., Negron K., Kim N., Garrison W.J., Miller G.W., Kingsmore K.M., Thim E.A., Song J., Munson J.M. (2020). Augmentation of brain tumor interstitial flow via focused ultrasound promotes brain-penetrating nanoparticle dispersion and transfection. Sci. Adv..

[146] Meng Y., Reilly R.M., Pezo R.C., Trudeau M., Sahgal A., Singnurkar A., Perry J., Myrehaug S., Pople C.B., Davidson B. (2021). MR-guided focused ultrasound enhances delivery of trastuzumab to her2-positive brain metastases. Sci. Transl. Med..

[147] Xiao N., Liu J., Liao L., Sun J., Jin W., Shu X. (2019). Ultrasound Combined with Microbubbles Increase the Delivery of Doxorubicin by Reducing the Interstitial Fluid Pressure. Ultrasound Q..

[148] Zhang Q., Jin H., Chen L., Chen Q., He Y., Yang Y., Ma S., Xiao S., Xi F., Luo Q. (2019). Effect of Ultrasound Combined With Microbubble Therapy on Interstitial Fluid Pressure and VX2 Tumor Structure in Rabbit. Front. Pharmacol..

[149] Eisenbrey J.R., Shraim R., Liu J.-B., Li J., Stanczak M., Oeffinger B., Leeper D.B., Keith S.W., Jablonowski L.J., Forsberg F. (2018). Sensitization of hypoxic tumors to radiation therapy using ultrasound-sensitive oxygen microbubbles. Int. Res. J. Biotechnol..

[150] Ho Y.J., Chu S.W., Liao E.C., Fan C.H., Chan H.L., Wei K.C., Yeh C.K. (2019). Normalization of Tumor Vasculature by Oxygen Microbubbles with Ultrasound. Theranostics.

[151] Chin C.T., Raju B.I., Shevchenko T., Klibanov A.L. Control and Reversal of Tumor Growth by Ultrasound Activated Microbubbles. Proceedings of the 2009 IEEE International Ultrasonics. Symposium.

[152] Burke C.W., Klibanov A.L., Sheehan J.P., Price R.J. (2011). Inhibition of glioma growth by microbubble activation in a subcutaneous model using low duty cycle ultrasound without significant heating. J. Neurosurg..

[153] Daecher A., Stanczak M., Liu J.B., Zhang J., Du S., Forsberg F., Leeper D.B., Eisenbrey J.R. (2017). Localized microbubble cavitation-based antivascular therapy for improving HCC treatment response to radiotherapy. Cancer Lett..

[154] Goertz D.E., Karshafian R., Hynynen K. Investigating the Effects of Pulsed Low Intensity Ultrasound and Microbubbles in Mouse Tumors. Proceedings of the 2009 IEEE International Ultrasonics. Symposium.

[155] El Kaffas A., Gangeh M.J., Farhat G., Tran W.T., Hashim A., Giles A., Czarnota G.J. (2018). Tumour Vascular Shutdown and Cell Death Following Ultrasound-Microbubble Enhanced Radiation Therapy. Theranostics.

[156] Skalina K.A., Singh S., Chavez C.G., Macian F., Guha C. (2019). Low intensity focused ultrasound (lofu)-mediated acoustic immune priming and ablative radiation therapy for in situ tumor vaccines. Sci. Rep..

[157] Hunt S.J., Gade T., Soulen M.C., Pickup S., Sehgal C.M. (2015). Antivascular ultrasound therapy: Magnetic resonance imaging validation and activation of the immune response in murine melanoma. J. Ultrasound Med..

[158] Jahangiri S., Stagg J., Yu F. UTMC Effect on Cancer Cell Apoptosis, Proliferation, and Vascular Inflammation in Wild Type and CD39 Knock Out Mice Model of MC38 Colon Cancer. Proceedings of the 2023 IEEE International Ultrasonics. Symposium.

[159] Lin L., Du Y., Hao J., Wu R., Du L. (2023). UTMD inhibits pancreatic cancer growth and metastasis by inducing macrophage polarization and vessel normalization. Biomed. Pharmacother..

[160] Joiner J.B., Kren N.P., Durham P.G., McRee A.J., Dayton P.A., Pylayeva-Gupta Y. (2022). Low-Intensity Focused Ultrasound Produces Immune Response in Pancreatic Cancer. Ultrasound Med. Biol..

[161] Liu H.L., Hsieh H.Y., Lu L.A., Kang C.W., Wu M.F., Lin C.Y. (2012). Low-pressure pulsed focused ultrasound with microbubbles promotes an anticancer immunological response. J. Transl. Med..

[162] Curley C.T., Stevens A.D., Mathew A.S., Stasiak K., Garrison W.J., Miller G.W., Sheybani N.D., Engelhard V.H., Bullock T.N.J., Price R.J. (2020). Immunomodulation of intracranial melanoma in response to blood-tumor barrier opening with focused ultrasound. Theranostics.

[163] Escors D. (2014). Tumour immunogenicity, antigen presentation and immunological barriers in cancer immunotherapy. New J. Sci..

[164] Dai Q., Wilhelm S., Ding D., Syed A.M., Sindhwani S., Zhang Y., Chen Y.Y., MacMillan P., Chan W.C.W. (2018). Quantifying the Ligand-Coated Nanoparticle Delivery to Cancer Cells in Solid Tumors. ACS Nano.

[165] Deprez J., Lajoinie G., Engelen Y., De Smedt S.C., Lentacker I. (2021). Opening doors with ultrasound and microbubbles: Beating biological barriers to promote drug delivery. Adv. Drug Deliv. Rev..

[166] Bulner S., Prodeus A., Gariepy J., Hynynen K., Goertz D.E. (2019). Enhancing Checkpoint Inhibitor Therapy with Ultrasound Stimulated Microbubbles. Ultrasound Med. Biol..

[167] Vos H., Lambein K., Richard F., Marien B., Nevelsteen I., Punie K., Wildiers H., Berben L., Laenen A., Floris G. (2021). Comparison of the tumor immune microenvironment of primary hormone receptor-negative HER2-positive and triple negative breast cancer. NPJ Breast Cancer.

[168] Voorwerk L., Sanders J., Keusters M.S., Balduzzi S., Cornelissen S., Duijst M., Lips E.H., Sonke G.S., Linn S.C., Horlings H.M. (2023). Immune landscape of breast tumors with low and intermediate estrogen receptor expression. NPJ Breast Cancer.

[169] Masih K.E., Wei J.S., Milewski D., Khan J. (2021). Exploring and Targeting the Tumor Immune Microenvironment of Neuroblastoma. J. Cell. Immunol..

[170] Zhang C., Guo L., Su Z., Luo N., Tan Y., Xu P., Ye L., Tong S., Liu H., Li X. (2021). Tumor Immune Microenvironment Landscape in Glioma Identifies a Prognostic and Immunotherapeutic Signature. Front. Cell Dev. Biol..

[171] Yang B., Li X., Zhang W., Fan J., Zhou Y., Li W., Yin J., Yang X., Guo E., Li X. (2022). Spatial heterogeneity of infiltrating T cells in high-grade serous ovarian cancer revealed by multi-omics analysis. Cell Rep. Med..

[172] Karpinski P., Rossowska J., Sasiadek M.M. (2017). Immunological landscape of consensus clusters in colorectal cancer. Oncotarget.

[173] Kurebayashi Y., Ojima H., Tsujikawa H., Kubota N., Maehara J., Abe Y., Kitago M., Shinoda M., Kitagawa Y., Sakamoto M. (2018). Landscape of immune microenvironment in hepatocellular carcinoma and its additional impact on histological and molecular classification. Hepatology.

[174] Chavez M., Silvestrini M.T., Ingham E.S., Fite B.Z., Mahakian L.M., Tam S.M., Ilovitsh A., Monjazeb A.M., Murphy W.J., Hubbard N.E. (2018). Distinct immune signatures in directly treated and distant tumors result from TLR adjuvants and focal ablation. Theranostics.

[175] Abe S., Nagata H., Crosby E.J., Inoue Y., Kaneko K., Liu C.X., Yang X., Wang T., Acharya C.R., Agarwal P. (2022). Combination of ultrasound-based mechanical disruption of tumor with immune checkpoint blockade modifies tumor microenvironment and augments systemic antitumor immunity. J. Immunother. Cancer.

[176] Goertz D., Cruz W., Bulner S., Wright A., Kerbel R. (2020). Abstract lb-079: Microbubble mediated focused ultrasound therapy enhances the antitumor potency and durability of anti-PD-L1 checkpoint blockade. Cancer Res..

[177] Wu N., Cao Y., Liu Y., Zhou Y., He H., Tang R., Wan L., Wang C., Xiong X., Zhong L. (2023). Low-intensity focused ultrasound targeted microbubble destruction reduces tumor blood supply and sensitizes anti-PD-L1 immunotherapy. Front. Bioeng. Biotechnol..

[178] Mouratidis P.X., Costa M., Rivens I., Repasky E.E., Ter Haar G. (2021). Pulsed focused ultrasound can improve the anti-cancer effects of immune checkpoint inhibitors in murine pancreatic cancer. J. R. Soc. Interface.

[179] Sheybani N.D., Breza V.R., Paul S., McCauley K.S., Berr S.S., Miller G.W., Neumann K.D., Price R.J. (2021). ImmunoPET-informed sequence for focused ultrasound-targeted mCD47 blockade controls glioma. J. Control Release.

[180] Tang J., Tang J., Li H., Zhou J., Tang N., Zhu Q., Wang X., Zhu B., Li N., Liu Z. (2023). Mechanical destruction using a minimally invasive Ultrasound Needle induces anti-tumor immune responses and synergizes with the anti-PD-L1 blockade. Cancer Lett..

[181] Tan X., Yi C., Zhang Y., Tang N., Xu Y., Liu Z. (2021). Ultrasound-Targeted Microbubble Destruction Alleviates Immunosuppression Induced by cd71(+) Erythroid Progenitor Cells and Promotes PDL-1 Blockade Immunotherapy in the Lewis Lung Cancer Model. Front. Oncol..

[182] Grzywa T.M., Justyniarska M., Nowis D., Golab J. (2021). Tumor Immune Evasion Induced by Dysregulation of Erythroid Progenitor Cells Development. Cancers.

[183] Chen J., Qiao Y.D., Li X., Xu J.L., Ye Q.J., Jiang N., Zhang H., Wu X.Y. (2021). Intratumoral cd45(+)cd71(+) erythroid cells induce immune tolerance and predict tumor recurrence in hepatocellular carcinoma. Cancer Lett..

[184] Sundaram K.M., Chang S.S., Penson D.F., Arora S. (2017). Decision Making as a Growth Mechanism in Interventional Oncology: Therapeutic Ultrasound and Prostate Cancer. Semin. Interv. Radiol..

[185] Xu Z., Khokhlova T.D., Cho C.S., Khokhlova V.A. (2024). Histotripsy: A Method for Mechanical Tissue Ablation with Ultrasound. Annu. Rev. Biomed. Eng..

[186] Rao R., Patel A., Hanchate K., Robinson E., Edwards A., Shah S., Higgins D., Haworth K.J., Lucke-Wold B., Pomeranz Krummel D. (2023). Advances in Focused Ultrasound for the Treatment of Brain Tumors. Tomography.

[187] Siedek F., Yeo S.Y., Heijman E., Grinstein O., Bratke G., Heneweer C., Puesken M., Persigehl T., Maintz D., Grüll H. (2019). Magnetic resonance-guided high-intensity focused ultrasound (mr-hifu): Technical background and overview of current clinical applications (Part 1). RöFo-Fortschritte auf dem Gebiet der Röntgenstrahlen und der Bildgebenden Verfahren.

[188] Romano G., Ventura E., Abraham K.S., Cersosimo F., Giordano A. (2022). Focused ultrasound therapy in cancer care. Ann. Res. Oncol..

[189] Kooiman K., Roovers S., Langeveld S.A.G., Kleven R.T., Dewitte H., O’Reilly M.A., Escoffre J.M., Bouakaz A., Verweij M.D., Hynynen K. (2020). Ultrasound-Responsive Cavitation Nuclei for Therapy and Drug Delivery. Ultrasound Med. Biol..

[190] Samanta D., McRae S., Cooper B., Hu Y., Emrick T., Pratt J., Charles S.A. (2008). End-Functionalized Phosphorylcholine Methacrylates and Their Use in Protein Conjugation. Biomacromolecules.

[191] Kwon M., Jung H., Nam G.-H., Kim I.-S. (2021). The right Timing, right combination, right sequence, and right delivery for Cancer immunotherapy. J. Control Release.

[192] DuPage M., Jacks T. (2013). Genetically engineered mouse models of cancer reveal new insights about the antitumor immune response. Curr. Opin. Allergy Clin. Immunol..

[193] DuPage M., Cheung A.F., Mazumdar C., Winslow M.M., Bronson R., Schmidt L.M., Crowley D., Chen J., Jacks T. (2011). Endogenous T cell responses to antigens expressed in lung adenocarcinomas delay malignant tumor progression. Cancer Cell.

[194] Garbe A.I., Vermeer B., Gamrekelashvili J., Wasielewski R.V., Greten F.R., Westendorf A.M., Buer J., Schmid R.M., Manns M.P., Korangy F. (2006). Genetically induced pancreatic adenocarcinoma is highly immunogenic and causes spontaneous tumor-specific immune responses. Cancer Res..

