# Supplementary material for: Fundamentals and Applications of Focused Ultrasound-Assisted Cancer Immune Checkpoint Inhibition for Solid Tumors
Source: Pharmaceutics. 2024 Mar 16;16(3):411. doi: 10.3390/pharmaceutics16030411 (PMC10976081; doi:10.3390/pharmaceutics16030411)
Supplement: Supplementary file 1 [file pharmaceutics-16-00411-s001.zip › pharmaceutics-2884340-supplementary.pdf]

Supplementary Materials: Fundamentals and Applications of Focused Ultrasound-Assisted Cancer Immune Checkpoint Inhibition for Solid Tumors

Sepideh Jahangiri and François Yu

Table S1. Summary of bioeffects following targeted FUS with/without MB treatment

	FUS modality	MB	I_{SPTA} (W/cm2)	F (MHz)	P (MPa)	PRF (kHz)	DC (%)	MI	ICI	Effect	Tumor type	Immune Phenotype
**Abe, 2022 [176]**	M-HIFU, T-HIFU	No	104, 988	1.5	12.5, 7.7	0.005, 0.001	2, 50	10.2, 6.3	aPD-L1	Greater systemic antitumor immune responses in M-HIFU (compared with T-HIFU), M1 macrophage polarization, M-HIFU+aPDL1 treatment increased CTLs and augmented abscopal effect. Major roles attributed to CTLs and NK cells in antitumor immunity. Tumor debulking in both modalities, extended survival and abscopal effect only in M-HIFU.	E0771, MM3MG-HER2	Immune excluded
**Bandyopadhyay, 2016 [118]**	M-HIFU, pFUS	No	734, 215	1	5.4, 2.9		75	5.4, 2.9	No	Tumor debulking, increased tumoral CD45+ but not CTLs, reversion of T cell anergy and tolerance, reduced pulmonary metastases and prolonged survival. HIFU is more effective if combined with LoFU prior to ablation.	B16F1, B16F10	Inflamed
**Chin, 2009 [151]**	M-HIFU	Yes		1.2	5	0.001, 0.00005		4.5	No	Transient vascular shutdown (up to 10 minutes), inflammatory response, tumor debulking,	MC38	Immune excluded
**Eranki, 2020 [96]**	M-HIFU	No		1.5	14	0.001		11.4	aCTLA-4, aPD-L1	Vascular shutdown, 62% improvement in survival in combined group, improved abscopal effect, increased TILs, systemic inflammation induction, overcome treatment resistance, tumor debulking.	Neuro2a	Immune excluded

**Iwanicki, 2023 [95]**	M-HIFU	No		1	35	0.05		35	No	Increased apoptosis and TNF-α, reduced pericyte coverage and PDGF-β, reduced hypoxia and VEGF. Vasodilation around ablated tissue.	NGP-Luc	Immune excluded
**Mouratidis, 2021 [179]**	M-HIFU	No	96	1.5	17	0.0001	1	13.9	aPD-1, aCTLA-4	Extended survival and tumor debulking, increased CTL	KPC	Immune excluded
**Pepple, 2023 [98]**	M-HIFU	No			30	0.1			CTLA-4	Inhibited tumor growth with abscopal effect, HMGB1 expression, expression of immunogenic cell death mediators and innate immune activation following increased NK cell infiltration, delayed tumoral CTL infiltration accompanied with activation of ferroptosis pathway in cancer cells. Maximal activation of ferroptosis when M-HIFU is combined with CTLA-4 blockade.	B16F10, Hepa1-6	Inflamed Immune excluded
**Qu, 2020 [92]**	M-HIFU	No		1	30	0.1		30	aCTLA-4	Tumoral and systemic TAA-lymphocyte responses, DAMP release (IFN-γ, HMGB1 and positioning of Calreticulin in membrane), histotripsy increased CTL infiltration greater than thermal ablative and 15Gy radiotherapy, abscopal effect, improved efficacy of aCTLA4 by histotripsy, tumor debulking	B16GP33, Hepa1-6	Inflamed and immune excluded, resp.
**Schade, 2019 [97]**	M-HIFU	No		1.5	17-20	0.001		13.8-16.3	No	TILs increase 2 days post treatment, vascular shutdown, systemic and intrarenal acute inflammatory responses (TNF-α, IFN-γ, HMGB1, IL-10 and IL-6 elevation)	Eker, rat model of renal carcinoma cell	Immune excluded
**Fite, 2021 [84]**	M-HIFU, T-HIFU	No	48, 320	3	16.9, 3.1		0.5, 100	9.7, 1.8	aPD-1 +/- CpG priming	Both modalities upregulate innate immune receptors (i.e. Nod1, Nlrp3, Aim2, TLR, and Cstb). Activation of wound healing inflammatory response. Upregulation of IL-1b and IL-6. CTL infiltration only increased in T-HIFU group when combined with aPD-1.	NDL	Immune excluded

**Chavez, 2018 [175]**	T-HIFU	No		3	3.1			1.8	aPD-1	100% tumor rejection when TLR9 agonist (CpG) and ablative FUS are combined (in NDL group). Increased T cell activation and infiltration increased IFN-γ, Macrophage and DC polarization toward CD169+ subset.	B16F10, NDL, MMTV-PyMT	Inflamed, immune excluded and immune excluded, resp.
**Deng, 2010 [83]**	T-HIFU	No		9.5					No	Trained DCs with HIFU-treated tumor, tumor lysate and mouse serum were studied. Reduced tumor growth in HIFU-ablated tumor DCs; increased DC response and IFN- and IL-12 in HIFU-treated tumor and tumor lysate groups. No difference in survival rate.	H22	Inflamed
**Sheybani, 2020 [112]**	T-HIFU	No		3			100		Gemcitabine +/- aPD-1	T-HIFU+ gemcitabine reduced tumor growth and increased survival. Better response while aPD-1 was administered earlier. Extended survival and tumor debulking.	4T1, E0771	Immune excluded
**Silvestrini, 2017 [85]**	T-HIFU	No		3	3.1			1.8	aPD-1	Vascular shutdown, primed immunotherapy strengthened FUS treatment, reduced immunosuppressive mediators while increased immunostimulatory cells, acute tumor debulking, HMGB1 increased, enhanced vascularization within the TDLNs (tumor draining lymph nodes), increased lymphatic drainage, complete response in 80%	NDL	Immune excluded
**Kheirolomoom, 2019 [75]**	HT	No		1.5	2.5	0.1		2	aPD-1 + CpG	Cupper-doxorubicin thermosensitive liposomes improved efficacy of immune-thermal combined treatment, 90% of mice rejected the tumor, increased TILs	B16F10, NDL, MMTV-PyMT	Inflamed, immune excluded and immune excluded, resp.
**Suzuki, 2010 [101]**	HT	Yes	0.7	1	0.7			0.7	No	IL-12 delivery, T cell infiltration, tumor debulking, vascular permeability	OV-HM	Immune desert

**Aydin, 2019 [76]**	pFUS,	No	3,<br>13,<br>53,<br>120,<br>213	1	1,<br>2,<br>4,<br>6,<br>8	10	10	1,<br>2,<br>4,<br>6,<br>8	No	Induced DNA strand breaks at PNP>6Mpa, anti-inflammatory responses suppressed at >4MPa, increased DAMPS and vascular inflammation at 6MP	4T1 and B16	Immune excluded and Inflamed, resp.
**Cohen, 2021 [77]**	pFUS,	No	120	1.15	6	0.005	10	5.6	No	Demonstrate tumor-type importance in defining FUS mediated antitumor immune responses, increased DAMPS, elevated CC3 up to 3 days, tumor debulking	4T1 and B16	Immune excluded and Inflamed, resp.
**Hayashi, 2022 [117]**	pFUS	No	3.0	3		0.001, 0.01, 0.1	20		aPD-1	Tumor debulking, increased CC3 and CD3-positive cells	TRAMP-C2	Immune desert
**Lee, 2017 [116]**	pFUS		42,<br>167	1.5	5,<br>10	0.001	5	4,<br>8.1	No	Tumoral ECM re-arrangement due to mechanical effects, increased deep penetration of chitosan nanoparticles	A549 (ECM-rich) and SCC7 (ECM-low)	Inflamed and immune desert, resp.
**Amate, 2019 [130]**	UTMC	Yes	12	1	0.85	0.2	50	0.85	aCD73	Increased mAb extravasation, effects of pulse length on drug delivery, vascular permeability	4T1	Immune excluded
**Belcik, 2017 [125]**	UTMC	Yes		1.3	1.5	9.3		1.3	No	Increased perfusion, reversed ischemia, increased eATP	-	-
**Bulner, 2019 [167]**	UTMC	Yes		1	1.65	1, 0.05		1.65	aPD-1	Vascular shutdown, enhanced efficacy of immunotherapy and tumor debulking	CT26	Immune excluded
**Burke, 2011 [152]**	UTMC	Yes	0.004	1	1.1	0.0002	0.01	1, 1.2	No	Vascular shutdown, tumor debulking, and apoptosis	C6	Immune desert

**Curley, 2020 [163]**	UTMC	Yes	0.04	1	0.5		0.5	0.5	No	Enhanced perfusion, BBB/BTB opening, vascular inflammation and cytokine release, increased infiltrated matured dendritic cells ($CD86^{+}$), increased TNF- , IL-6, CXCLs, I-CAM1 and PSMB88 (immunoproteasome subunit functioning in antigen presentation) at 6 and 24h post sonication.	B16F1-OVA	Inflamed
**Curley, 2020 [145]**	UTMC	Yes	0.03, 0.05	1.1	0.45, 0.55		0.5	0.43, 0.52	No	BBB and BTB opening augments the velocity and direction (~70-80°) of interstitial flow, increased tumoral dispersion of nanoparticles by >100%.	U87	Immune desert
**Daecher, 2017 [153]**	UTMC	Yes		4.2	2.5	0.04		1.2	No	Vascular shutdown, tumor debulking when combined with radiotherapy (5Gy)	Hu 7.5	Immune excluded
**El Kaffas, 2018 [155]**	UTMC	Yes	1.1	0.5	0.6	3	10	0.8	No	Vascular shutdown up to 72 h , cell death increased, improved radiotherapy	MCA-129	Immune desert
**Eisenbrey, 2018 [149]**	UTMC	Yes		4.2	2.5	0.004		1.4	No	Reduced hypoxia, O2 delivery, increased tumoral O2, increased radio-sensitization, tumor debulking	MDA-MB-231	Immune excluded
**Goertz, 2009 [150]**	UTMC	Yes	0.004	1	0.74	1, 0.05	0.024	0.7	No	Antivascular effects induced tumor growth delay	MeWo	Inflamed
**Goertz, 2020 [177]**	UTMC	Yes	0.06	0.6	3		0.02	3.9	aPD-L1	Vascular shutdown , complete tumor debulk in 7 out of 8 mice, increased survival	EMT6	Immune excluded
**Hunt, 2015 [156]**	UTMC	Yes	0.02	3	0.22		1	0.13	No	Vascular shutdown, increased hypoxia, increased CD45+/CD3+ cells	K1735	Inflamed
**Jahangiri, 2023 [158]**	UTMC	Yes	12	1	0.85	0.2	50	0.85	No	Perfusion maintenance, induced vascular inflammation, increased apoptosis and decreased cancer cell proliferation	MC38	Immune excluded

**Joiner, 2022 [160]**	UTMC	Yes	0.8	1	0.5	0.1	10	0.5	No	Single UTMC monotherapy reduced tumor growth by 2 weeks post treatment; no effect on TILs but increased APCs in TDLNs, no increase in HMGB1, CC3, and HSP70 indicating necessitate of repeating times of treatment or combining it with immunotherapy	KPC	Immune excluded
**Kovacs, 2017 [142]**	UTMC	Yes	0.03	0.6	0.3		1	0.4	No	BBB opening, increased DAMP, sterile inflammation, NF B signaling lasted for 24h, increased ICAM. Vascular inflammation.	-	
**Li, 2021 [90]**	UTMC	Yes	0.04, 0.4	4	0.8, 2.4	0.001	0.2	0.4, 1.2	aPD-L1	Increased tumor perfusion, tumor vascular normalization, tumor debulking, immune cell infiltration	MC38	Immune excluded
**Liu, 2012 [162]**	UTMC	Yes		0.5	0.6, 1.4	0.001		0.85	No	Vascular permeability, tumor debulking, continuous non-T_{reg} cell infiltration, increased mast cells at day 1 post treatment but decreased afterwards.	CT26	Immune excluded
**McMahon, 2017 [143]**	UTMC	Yes		0.6	0.3				No	High MB dose (10X) but not the clinical dose, induces NFκB signaling, sterile inflammation	-	
**Meng, 2021 [146]**	UTMC	Yes	0.001, 0.06	0.2	0.5		0.7	1	Trastuzumab	BBB opening, increased drug delivery, tumor debulking, no severe adverse side effect in human patients. Vascular permeability	HER2+ brain metastases	Immune excluded
**Michon, 2022 [79]**	UTMC	Yes		1, 1.3	0.25-0.75, 1.5				No	Reduced hypoxia, increased perfusion, reduced HIF1 expression, increased radiosensitization at 8 Gy	PC3	Immune desert
**Sabbagh, 2021 [144]**	UTMC	Yes	0.075	1	0.3	0.001	2.5	0.3	aPD-1	Increased median survival of UTMC+aPD1 group to 58 days vs. 39 days in control group. Increased delivery of CAR T-cell to the CNS after 24h and 72h post treatment. Increased median survival in combined vs. monotherapy of CAR T cell by 129%. Local increase in CXCL-10 secreting APCs and infiltrated CTLs in the TME	GL261	Immune desert

**Sheybani, 2021 [180]**	UTMC	Yes	0.03	1.1	0.4		0.5	0.4	aCD47	BBB opening enhances aCD47 delivery. Superlative delivery of aCD47 if it is injected after UTMC treatment. Repeated session of UTMC increased aCD47 delivery, tumor debulking and extended survival.	GL261	Immune desert
**Tan, 2021 [182]**	UTMC	Yes	0.1 - 0.115	5	2.19 - 2.30	0.5	0,065	0.98 - 1.03	aPD-L1	In the UTMC group, splenic CEC reduction and CTL enhancement; In the combined group, tumor growth inhibition, increased IFN-γ+ CTL and $CD4^+$ T cells, and reduction of TGF-β^+ $CD11b^+$ cells.	LLC	Inflamed
**Tang, 2023 [181]**	UTMC, T-HIFU	Yes		0.025		0.15	50, 100		aPD-L1	Enhanced therapeutic effect with UTMC in reducing tumor growth and improving survival rate. Tumor perfusion reduced 48h post UTMC, inflammatory responses in tumor and TDLNs increased two days post UTMC, increased calreticulin, HSP70, and HMGB1Increased TILs (CTL, CD4+Th17+, and TAM), decreased Treg and MDSCs two days post-UTMC.	MC38	Immune excluded
**Wu, 2023 [178]**	UTMC	Yes	3	1	0,4		50		aPD-L1	Blood vessel rupture caused perfusion shut down, tumor growth inhibition, induced ICD due to increased calreticulin, increased DC and CTL in tumor and TDLNs, enhanced IL-12 and TNF-α expression, improved aPD-L1 efficiency following combined treatment.	4T1	Immune excluded
**Xiao, 2019 [147]**	UTMC	Yes	0.1	1	1	0.01	0.2	1	No	Vascular permeability, reduced IFP and improved drug penetration in tumors	VX2	Immune excluded
**Zhang, 2019 [148]**	UTMC	Yes	0.07, 0.6, 1.7	1	1, 3, 5	0.01	0.2	1, 3, 5	No	Reduced IFP at 3 and 5 MPa but no changes in the morphology of collagen and reticular fibers in rabbit tumors 24h post treatment	VX2	Immune excluded

DC%: Duty Cycle; F: Frequency; HT: Hyperthermia; ICI: Immune Checkpoint Inhibitor; I_{SPTA}: Spatial peak temporal average intensity; M-HIFU: Mechanical HIFU; MI: Mechanical Index; P: pressure; pFUS: pulsed FUS; PRF: Pulse Repetition Frequency; T-HIFU: Thermal HIFU; UTMC: Ultrasound Targeted Microbubble Cavitation.
